# A Preliminary Food–AI Readiness Framework for AI-Enabled Hyperspectral Systems in Fungal and Mycotoxin Risk Management: A Critical Narrative Review

**DOI:** 10.3390/foods15162860

**Published:** 2026-08-17

**Authors:** Ömer Faruk Yeşil

**Affiliations:** Department of Food Processing, Diyarbakır Vocational School of Agriculture, Dicle University, 21280 Diyarbakır, Türkiye; faruk@dicle.edu.tr

**Keywords:** artificial intelligence, hyperspectral imaging, mycotoxins, food safety, decision support

## Abstract

Fungal contamination and mycotoxins remain important hazards in cereal, nut, oilseed, and processed-food chains. For grain handlers, processors, storage facilities, quality-assurance laboratories, and technology developers, the practical challenge is not only detection but determining when an AI-enabled optical output is sufficiently documented to support accepting, holding, sorting, or referring material for confirmatory analysis. This critical narrative review, supported by structured evidence appraisal, examines artificial intelligence (AI)-enabled hyperspectral imaging (HSI) for fungal and mycotoxin risk management. The focused evidence map comprises a combined 20-study evidence map, including 17 spectral-imaging studies and three contextual non-imaging near-infrared studies addressing aflatoxins, fumonisins, deoxynivalenol (DON), zearalenone (ZEN), ochratoxin A (OTA), fungal status, and co-contamination; no patulin-specific AI-HSI study met the defined corpus criteria. Most evidence remains laboratory-based, with controlled contamination, limited independent validation, incomplete transfer testing, or no line-level demonstration. The review proposes the preliminary Food–AI Deployment-Readiness Framework (F-AIDRF), a non-regulatory appraisal tool covering analytical validity, algorithmic robustness, explainability and auditability, transferability and maintenance, operational feasibility, and decision integration. Applied descriptively, F-AIDRF is intended to reveal missing evidence, not to certify technologies. For food-industry readers, it offers a practical vocabulary for comparing screen-sort-confirm systems before investment, pilot testing, or regulatory-facing use.

## 1. Introduction

Fungal contamination and mycotoxins can enter the food chain during crop production, harvest, drying, storage, transport, processing, and retail. Cereals, nuts, oilseeds, spices, and their derivatives are vulnerable when environmental and handling conditions favor fungal growth or when postharvest control is inadequate. Aflatoxins, deoxynivalenol, zearalenone, fumonisins, and ochratoxin A are major concerns because of their health and economic consequences [1,2].

Contamination is uneven and strongly context-dependent. Genotype, season, temperature, humidity, water activity, pest damage, storage duration, geographical origin, and processing conditions influence fungal growth and toxin accumulation. In maize, a small fraction of kernels may carry much of the contamination; bulk tests can miss high-risk units, whereas a few heavily contaminated kernels can determine the fate of an entire lot [3]. Effective risk management therefore requires representative screening and confirmation before suspect material moves further along the chain.

HPLC, LC–MS/MS, GC–MS, ELISA, and immunoaffinity methods remain reference tools for confirmatory mycotoxin analysis. They are sensitive, selective, and relevant to regulatory decisions, but they require representative sampling, homogenization, extraction, laboratory infrastructure, trained personnel, and time. These requirements limit their use for high-throughput sorting and immediate decisions [2,4]. Optical systems should therefore complement, not replace, reference analysis by directing suspect material to confirmation, segregation, or targeted intervention.

HSI is a non-destructive technique that combines spatial information with wavelength-dependent spectra. Depending on the platform, it records patterns linked to moisture, composition, fungal colonization, structural damage, oxidation, or contamination-associated matrix changes across VIS–NIR, SWIR, fluorescence, Raman, or multispectral ranges [1,5].

In this context, artificial intelligence is not merely a statistical add-on to hyperspectral imaging. It determines how high-dimensional spectral–spatial data are converted into food-safety categories, concentration estimates, uncertainty signals, or operational actions. AI models can learn contamination-associated patterns, but they may also learn batch effects, illumination differences, cultivar-specific features, sample-presentation artifacts, or other unstable correlations. The central question is therefore not whether an algorithm can achieve high accuracy in a development dataset, but whether the complete AI-HSI pipeline can support a defensible food-safety decision under changing matrices, instruments, seasons, and operating conditions.

In this review, AI is used as an umbrella term for data-driven computational modeling, including chemometrics, statistical learning, conventional machine learning, deep learning, transfer learning, and explanation methods. This wording does not imply that every method is autonomous or general-purpose AI. Many retained studies use established statistical-learning or chemometric algorithms; they are included because contemporary food-safety practice increasingly evaluates them within AI-enabled sensing pipelines.

AI-enabled HSI should therefore be evaluated as a learning and decision pipeline. Relevant evidence extends from sample selection and reference-method alignment to preprocessing, feature extraction or representation learning, model training, threshold setting, uncertainty handling, and post-deployment monitoring. A model trained on weakly aligned labels or validated only through internal random splits may appear technically successful while remaining unsuitable for screening, sorting, or regulatory triage. Conversely, a simpler but well-validated model may be more useful if it controls false negatives, remains stable across domains, and provides outputs that can be audited and linked to confirmatory analysis.

More spectral data do not, by themselves, solve a food-safety problem. Spectra are affected by orientation, cultivar, texture, particle size, moisture, storage history, illumination, temperature, sensor noise, and device-specific effects. Models may therefore detect indirect effects of fungal colonization rather than the toxin molecule itself and can fail when challenged with natural contamination, a new season, another instrument, or a moving line.

Accuracy alone is a poor proxy for food-safety utility. A model can score highly while missing contaminated samples when prevalence is low or classes are imbalanced. False negatives may release contaminated material, whereas excessive false positives increase waste and cost. Sensitivity, specificity, precision, recall, F1-score, AUC, false-negative rate, prediction bias, and threshold uncertainty should therefore be interpreted together [6,7].

Transferability separates laboratory feasibility from dependable use. Performance can change with the instrument, commodity, contamination mechanism, or operating environment. Deng et al. [4] found direct transfer across grain-toxin tasks unreliable, although target-domain correction improved task-specific predictions; the study did not establish an instrument-to-instrument deployment protocol. Dependable use requires monitoring, recalibration, external evaluation, and clear referral rules for confirmation.

**Figure 1:**
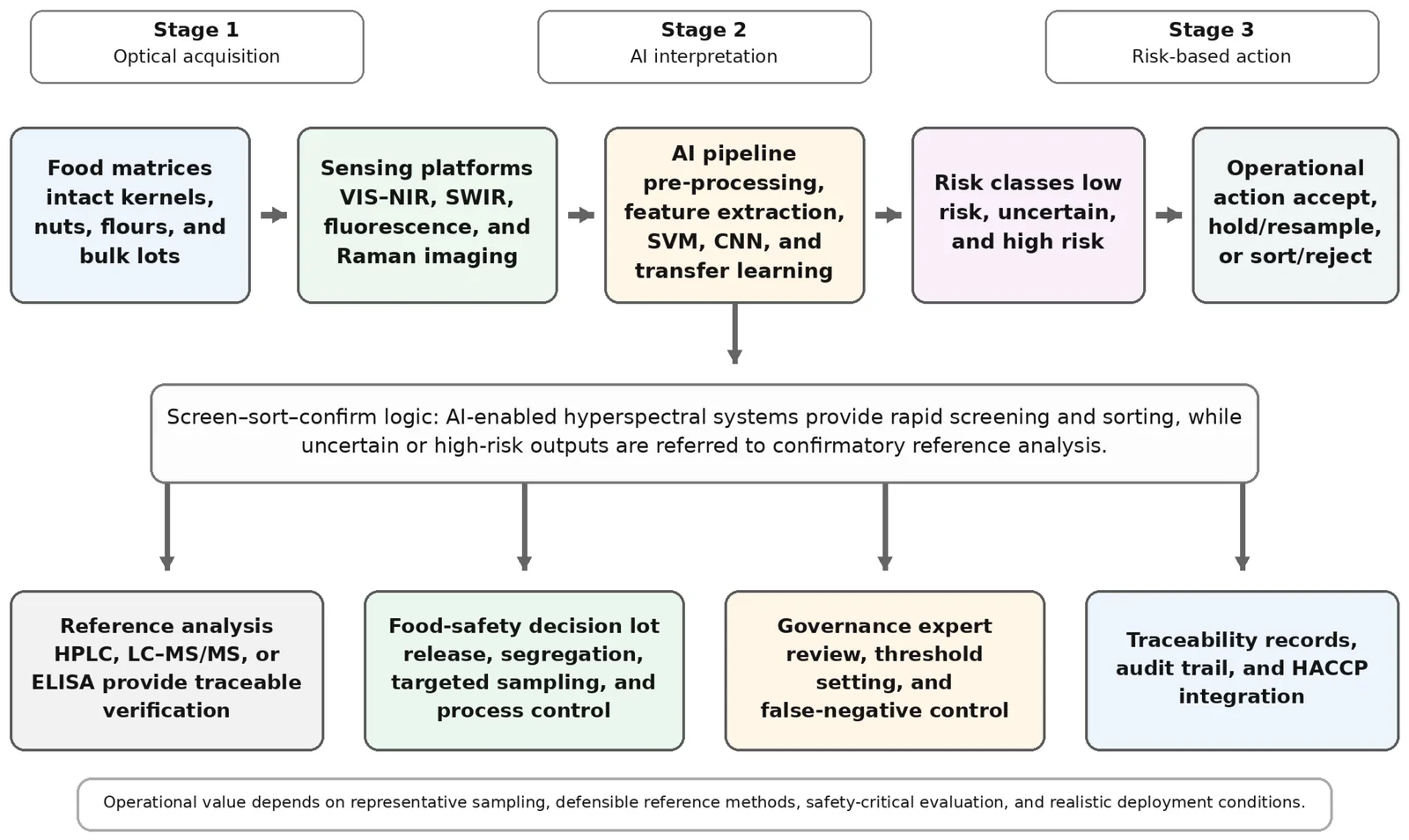
Three-stage screen–sort–confirm workflow for AI-enabled hyperspectral food-safety decisions. Abbreviations: VIS–NIR, visible and near-infrared; SWIR, short-wave infrared; SVM, support vector machine; CNN, convolutional neural network.

This review synthesizes AI-enabled HSI for fungal and mycotoxin risk assessment; compares platforms, processing strategies, and model architectures; examines safety-critical performance, explainability, transferability, and model-lifecycle requirements; and proposes a preliminary F-AIDRF. The review is intended for food-industry readers, analytical laboratories, equipment developers, and food-safety researchers who must decide whether an optical AI result is merely promising or sufficiently documented to justify screening, sorting, confirmatory referral, or pilot-line testing. Section 2 introduces the framework logic, Section 9 applies it, and neither section presents it as a validated standard. Figure 1 shows the screen–sort–confirm logic that connects matrix-specific acquisition and AI interpretation with risk triage and confirmatory referral.

Stage 1 acquires spectra from kernels, nuts, powders, or bulk lots. Stage 2 applies preprocessing, feature extraction, and AI-based classification or prediction. Stage 3 translates the output into acceptance, a temporary hold with resampling or intensified review, or sorting/rejection followed by HPLC or LC–MS/MS when required.

## 2. Review Type, Scope, and Structured Evidence Appraisal

### 2.1. Review Type, Scope, and Evidence Selection

This manuscript is a critical narrative review supported by structured evidence appraisal. It was not designed as a protocol-registered systematic review and does not claim PRISMA compliance, pooled effect estimation, dual-reviewer screening, or exhaustive retrieval. The evidence map is a focused analytical corpus assembled to support critical interpretation of deployment-readiness evidence, not a census of all AI and spectral-sensing studies.

The final evidence-identification checks were conducted up to 1 July 2026, with no lower date limit. Records were located or verified through Web of Science, Scopus, PubMed, ScienceDirect or publisher repositories, and official regulatory sources. Duplicate records were removed manually by matching title, DOI, authorship, year, and journal. Candidate records were screened by title/abstract and then by full text for endpoint, sensing modality, food or feed matrix, reference target, and modeling approach. Table 1 and Supplementary Table S2 document the search logic, selection boundaries, and exclusion categories.

Eligible primary evidence comprised English-language, peer-reviewed spectral-imaging studies with a predictive or classification model linked to a traceable chemical, molecular, microbiological, visual, or validated experimental reference. Three non-imaging NIR studies were retained only as contextual evidence for transferability or portability and were not interpreted as HSI-equivalent validation. Reviews and methods papers provided context for analytical validity, explainability, transferability, and reporting. General quality studies, non-food matrices, descriptive spectroscopy without a predictive model, and reports without an identifiable reference target were excluded. The focused map comprises a combined 20-study evidence map consisting of 17 spectral-imaging studies and 3 contextual non-imaging NIR studies, with 5 reviews, 7 methodological papers, and EU, Codex, and FDA regulatory or guidance sources retained for context. No patulin-specific AI-HSI primary study met the defined corpus criteria; this is a corpus-boundary statement rather than a general conclusion about the entire field.

### 2.2. Critical Appraisal, Evidence Profile, and Framework Derivation

For each primary study, the appraisal recorded endpoint category, optical measurement unit, reference-analysis unit, unit alignment or mismatch, contamination origin, sensor and wavelength configuration, preprocessing, model type, data partitioning and validation, leakage-control status when reportable, safety-critical metrics, explainability, transferability, operational relevance, and the provisional F-AIDRF stage. Supplementary Table S1 reports these study-level evidence profiles. The appraisal is a single-author extraction of reported information; it is not an independent replication, inter-rater validation, or formal risk-of-bias score.

F-AIDRF coding followed explicit decision rules, now expanded as a structured checklist in Supplementary Table S3. Each domain contains mandatory criteria, desirable criteria, evidence examples, and adjudication rules. Missing or unclear evidence was coded conservatively as “not reported/unclear” and could not be used to advance readiness. The six domains were treated as mandatory at the domain level for progression beyond R2, but the checklist distinguishes criteria required for advancement from desirable evidence that strengthens, but does not by itself determine, a stage assignment. Thus, auditability records are mandatory for high-stakes use, whereas post hoc XAI evidence is desirable and use-case dependent; similarly, quantitative operational-feasibility information is mandatory for R4+ pilot-line claims, whereas cost-of-ownership, downtime, environmental-tolerance, and computing-load details are desirable supporting evidence. The minimum evidence required in each domain depends on the intended use: laboratory method development, screening, sorting, hold/release triage, or confirmatory-referral decision support.

Readiness stages are introduced here to prevent confusion later in the manuscript. R0 denotes evidence that is not deployment-assessable; R1 denotes laboratory proof-of-concept; R2 denotes an analytical screening candidate with traceable reference evidence and basic threshold or error reporting; R3 requires independent external or cross-domain decision-support evidence; R4 requires pilot-line or moving-product evidence; and R5 denotes controlled routine implementation evidence with governance and confirmation. Borderline cases remain at the lower stage unless the mandatory criteria for the higher stage are explicitly reported. These stages are provisional descriptors, not empirical cut-offs, certifications, or regulatory categories.

Because one author conducted the coding, the appraisal is intentionally conservative and should be read as a transparent, descriptive coding exercise rather than as an independently validated risk-of-bias assessment. The absence of a second assessor is a substantive limitation: readiness-stage assignment requires judgment, particularly when studies incompletely report reference-unit alignment, leakage control, external validation, or operational evidence. To reduce over-interpretation, unclear information was never treated as positive evidence, borderline cases were retained at the lower stage, and all study-level decisions were documented in Supplementary Tables S1 and S3. Nevertheless, independent reassessment remains necessary before F-AIDRF can be used as a validated appraisal instrument.

For reviewer transparency, the coding logic is therefore separated into three layers: extraction variables that describe what each study reported (Table 2 and Supplementary Table S1), provisional readiness stages assigned using explicit decision rules (Table 3 and Supplementary Table S3), and future validation steps needed to test whether independent users reach similar ratings (Section 11.2). This separation is intended to address possible circularity between framework development and descriptive application.

The profile reveals a major translational gap. The combined evidence map contains 20 study entries: 17 spectral-imaging studies and three contextual non-imaging NIR studies. When HSI-equivalent readiness staging is counted, the denominator is the 17 spectral-imaging studies: 13 were classified as R1 laboratory proof-of-concept evidence and four as R2 analytical screening candidates. The three non-imaging NIR entries are included in the combined 20-study evidence map only as contextual evidence for portability, threshold screening, or calibration-transfer interpretation and are not treated as HSI-equivalent stage evidence. None of the 20 entries met the conditions for R3–R5. Six entries mainly used naturally occurring contamination, ten used controlled, inoculated, or spiked material, and four used mixed, secondary, or incompletely described source designs. Three papers reported transferability evidence and five provided operational-feasibility evidence. These values describe the retained corpus and should not be interpreted as pooled estimates of technology performance.

The synthesis is therefore interpreted by analytical task rather than by the aggregate HSI-equivalent R1/R2 counts alone. The retained corpus separates into non-interchangeable task groups: fungal-status or infection/damage indicators [6,11,12,16,17]; aflatoxin-oriented threshold, concentration, or sorting studies in maize, nuts, or kernels [3,8,9,10,13,14,15,19,20,21,22]; co-contamination classification [7]; ZEN/OTA-oriented evidence [18]; and contextual non-imaging NIR portability or transfer studies [4,14,23]. These groups support different claims: fungal-status models can inform early warning, toxin models require chemically aligned reference values, kernel-level models are relevant to sorting, bulk-sample models are relevant to lot triage, and contextual NIR studies inform portability but cannot be interpreted as HSI deployment evidence.

### 2.3. Scope Boundaries and Interpretive Limits

This review prioritizes spectral imaging because spatial resolution is central to screening and unit-level sorting. FT-NIR, portable NIR, handheld Raman, electronic-nose, and smartphone systems may have complementary roles, but they were not reviewed comprehensively because their measurement units, signal structures, and constraints differ from HSI. They are discussed only as contextual evidence for transferability or portability.

The findings should not be generalized to all food-safety AI systems or all mycotoxins. Patulin, T-2/HT-2 toxins, ergot alkaloids, and other hazards remain important targets, but the retained evidence was too limited for technology-specific claims. This boundary is stated explicitly in the synthesis and research agenda.

## 3. Scientific and Analytical Foundations

### 3.1. Hazard–Matrix–Reference Alignment

Fungal contamination arises across preharvest, harvest, postharvest, storage, processing, and distribution stages. The hazard extends beyond visible mold: propagules, metabolic activity, structural damage, and mycotoxin residues may occur alone or together, depending on commodity, fungal species, environment, and food-chain stage. Cereals, nuts, oilseeds, spices, dried foods, and derivatives are vulnerable because moisture, nutrient composition, physical damage, and storage can create favorable niches for toxigenic fungi.

Fungal growth and mycotoxin occurrence are not linearly related. Visibly moldy food may be below a regulatory threshold, whereas apparently sound material may contain localized toxin residues or early infection. AI-enabled sensing may therefore capture indirect signals from colonization, moisture redistribution, biochemical degradation, structural damage, or endogenous fluorophores rather than the mycotoxin molecule itself.

Contamination heterogeneity intensifies food-chain risk. In maize, aflatoxin can be concentrated in a small subset of kernels; bulk analysis may miss rare positives, while a few heavily contaminated units can trigger rejection or downgrading of a lot. Individual-kernel inspection may enable targeted removal before mixing, milling, storage, or further processing [3].

Fungal and mycotoxin monitoring is a risk-management task that combines early warning, representative sampling, analytical confirmation, lot segregation, targeted rejection, and traceable decisions. AI-enabled HSI can support these actions at a speed and scale beyond routine laboratory analysis.

Reliable AI models require reliable ground truth. HPLC and LC–MS/MS provide chemical toxin values; ELISA and immunoaffinity assays may provide screening or confirmatory measurements depending on validation; and fungal DNA, spore counts, microbiological enumeration, and visual grades describe biological or phenotypic states rather than toxin concentration. These reference types are not interchangeable: a model trained against fungal status detects fungal status, not toxin concentration.

Minimum reference-quality reporting should include the sampling plan, the analytical unit, extraction recovery or method recovery where available, matrix effects, limit of detection, limit of quantification, repeatability or replicate analysis, calibration range, reference-method precision, use of certified reference material or validated quality-control material where relevant, and uncertainty near the regulatory or screening threshold. Poor AI performance may reflect weak optical information, but it may also reflect noisy or misaligned labels; conversely, high model performance can be misleading when reference labels are not analytically secure.

Fungal detection must remain distinct from toxin detection. A model trained on moldy kernels, fungal DNA, or spore burden may identify infection but cannot be treated as a predictor of regulatory mycotoxin concentration without an aligned chemical reference. Conversely, a model based on a bulk HPLC value may support lot-level risk assessment but is not an individual-kernel toxin measurement unless its reference design matches the imaged unit.

Sampling and preparation shape ground-truth quality. Grinding and homogenizing material before chemical analysis improve uniformity but can mask individual-kernel heterogeneity, whereas kernel-level HSI classifies individual units. Each imaged kernel should therefore be traceable to an individual or pooled reference measurement representing the same analytical unit.

Natural-contamination datasets capture realistic biological variation, storage effects, and complex contamination patterns, although they are difficult to obtain and may have imbalanced classes or narrow concentration ranges. Artificially inoculated or spiked samples remain useful for controlled development, but may differ spectrally from natural contamination; for aflatoxin, deposition differs between applied toxin solutions, laboratory inoculation, and natural contamination [3,8].

### 3.2. Optical Platforms, Data Structure, and Pre-Processing

Hyperspectral and multispectral systems differ in spectral range, resolution, acquisition mode, cost, data volume, and analytical suitability. Both convert food–light interactions into information linked to contamination, composition, or quality. The appropriate platform depends on the matrix, target hazard, contamination mechanism, required throughput, and decision context.

VIS–NIR imaging captures color, pigments, surface defects, moisture-related variation, fungal discoloration, and some structural features. SWIR provides stronger access to C–H, O–H, and N–H bond-related absorption patterns and can reveal changes in moisture, protein, lipid, carbohydrate, and fungal-induced biochemical composition. In food-safety applications, VIS–NIR and SWIR are often complementary [9,10].

Fluorescence HSI actively excites samples with ultraviolet, laser, or light-emitting diode sources, exploiting fluorescence from fungal metabolites, endogenous components, or contamination-related changes. Its high sensitivity can be limited by overlapping signals from native fluorophores; in maize, aflatoxin B_1_ fluorescence may overlap with aromatic amino acids, tocopherols, and other constituents [3].

Multispectral imaging uses selected bands rather than hundreds of continuous wavelengths, reducing hardware cost and computational load and easing in-line deployment. Its performance depends on robust wavelength selection. Chemically meaningful, stable bands may support more useful industrial systems than high-dimensional HSI that works only under laboratory conditions, whereas poor selection can reduce robustness across varieties, instruments, or contamination levels.

Platform choice should follow the intended decision. A system that rejects visibly abnormal kernels requires less spectral detail than one estimating toxin concentration near a regulatory threshold, and a laboratory platform optimized for characterization may be too slow for sorting. The relevant question is whether the sensor-model combination has adequate analytical validity for the intended action.

A hyperspectral image is a three-dimensional data cube with two spatial dimensions and one spectral dimension. Each pixel has a spectrum, and each wavelength band forms a spatial image. This structure shows both where a feature occurs and how it interacts with light, which is useful when contamination is localized on a surface, across a kernel, near damaged tissue, or in specific microstructural regions.

HSI’s richness also complicates analysis. A single image can contain hundreds of bands and thousands of pixels, creating correlated and redundant variables. Spectral responses are further affected by particle size, water activity, roughness, curvature, composition, seed-coat properties, cultivar, storage history, fungal colonization, and scattering; they may change even when toxin concentration does not.

Matrix complexity makes transfer across products and environments difficult. A classifier trained on one maize variety may use moisture or structural patterns that do not hold in another, and a model trained on uniform laboratory samples can fail on naturally contaminated commodities with different damage, fungal strains, storage histories, and background composition. These sources of domain shift explain why strong internal validation may not reproduce externally.

Preprocessing directly affects reliability. Raw hyperspectral data may contain noise, baseline shifts, scattering, illumination gradients, dead pixels, background signal, and physical variation unrelated to contamination. Appropriate preprocessing removes irrelevant variation while retaining spectral information associated with fungal contamination or mycotoxin risk.

Common preprocessing steps include smoothing, baseline correction, normalization, standard normal variate transformation, multiplicative scatter correction, Savitzky–Golay filtering, first- and second-derivative transformations, detrending, and wavelet denoising. Standard normal variate and multiplicative scatter correction reduce effects of particle size, scattering, path-length differences, and surface properties; derivatives can reveal subtle absorption features and reduce baseline effects but may amplify noise if poorly optimized [5].

Feature extraction and wavelength selection reduce the dimensionality of hyperspectral data. Principal component analysis, competitive adaptive reweighted sampling, successive projections algorithms, uninformative variable elimination, variable importance in projection, genetic algorithms, and related methods can reduce redundancy, limit overfitting, simplify multispectral hardware, and aid interpretation. A band selected in one small dataset may still fail to transfer across cultivars, seasons, instruments, or processing environments.

Table 4 summarizes the main sensing platforms, their constraints, and their most defensible food-safety roles.

### 3.3. AI Learning Architectures, Data Fusion, and Model Governance

An AI-enabled HSI system should be understood as a complete learning pipeline rather than as a single classifier. The pipeline begins with image acquisition and calibration, continues through background removal, segmentation, spectral preprocessing, feature extraction or representation learning, and ends with a decision rule that converts model output into a food-safety action. Weakness at any stage can compromise the final decision. For example, leakage introduced before train-test splitting, augmentation applied across related samples, or threshold selection based only on internal data can inflate apparent performance and reduce real-world reliability.

Conventional machine-learning and chemometric approaches remain central to hyperspectral food-safety assessment. Partial least squares regression, partial least squares discriminant analysis, linear discriminant analysis, support vector machines, random forests, k-nearest-neighbor classifiers, and artificial neural networks are widely used because they can perform well with moderate datasets and provide comparatively transparent modeling pathways. Their value is greatest when spectral features are carefully preprocessed, the reference target is clearly aligned with the imaged unit, and the objective is explicitly defined as binary classification, multiclass categorization, or concentration prediction [2,5].

Deep-learning methods extend this capability by learning non-linear spectral and spatial representations directly from the data. One-dimensional convolutional neural networks (CNNs) can model extracted spectra, whereas two-dimensional and three-dimensional CNNs can incorporate spatial texture and spectral relationships simultaneously. Recurrent neural networks, long short-term memory (LSTM) networks, gated recurrent units, attention mechanisms, and hybrid CNN-LSTM architectures may be useful where sequential spectral dependencies or multimodal information are important. These models can reduce the need for manual feature engineering, but they typically require larger and more diverse datasets, careful regularization, transparent reporting of architecture and hyperparameters, and validation that is independent of the training source.

The apparent power of AI also creates specific risks. Hyperspectral datasets often contain multiple spectra, pixels, regions of interest, or augmented images derived from the same biological sample. If related observations are distributed across training and test sets, the model may learn sample identity, imaging-session effects, or background variation rather than contamination-relevant information. Data splitting should therefore occur at the independent sample, lot, batch, harvest, or production-event level before feature engineering or augmentation. For food-safety decisions, model evaluation should also report sensitivity, false-negative counts, threshold behavior, and class-specific error patterns rather than only global accuracy.

Sensor and data fusion provide another route to improved robustness. Fusion can occur at pixel level, feature level, or decision level. Pixel-level fusion combines raw signals from different sensors; feature-level fusion combines extracted variables; and decision-level fusion integrates the outputs of separate models. In maize aflatoxin detection, dual-wavelength fluorescence data fusion improved prediction compared with individual spectral sources, demonstrating the value of complementary information when single-sensor signals are affected by interference or incomplete representation of contamination [3]. Fusion, however, should be justified by improved safety-critical performance and not merely by the availability of additional sensors.

AI outputs need to be translated into operational decisions. A model may produce class labels, class probabilities, risk scores, regression estimates, uncertainty intervals, or spatial heat maps, but none of these outputs is automatically a food-safety decision. The intended action must be specified: release, hold, sort, reject, resample, intensify inspection, or submit to confirmatory analysis. Decision thresholds should reflect the cost of false negatives, the burden of false positives, the uncertainty of the reference method, and the prevalence expected in routine monitoring. In this sense, AI model design and food-safety decision design are inseparable.

Model governance is equally important. AI-enabled HSI systems need version control for datasets, preprocessing routines, model weights, decision thresholds, calibration updates, and software changes. They also require monitoring for domain shift when matrices, cultivars, moisture conditions, instruments, lighting, operators, or production environments change. The transfer-learning evidence discussed in this review shows why portability or cross-task adaptation cannot be assumed to equal deployment readiness; target-domain correction may improve performance, but full source-to-target operational validation remains necessary [4].

The usefulness of AI-enabled hyperspectral systems therefore depends on six aligned elements: a representative food matrix, a defensible ground-truth method, a suitable sensing platform, appropriate preprocessing, transparent model development, and realistic validation. HSI is powerful because it captures rich spectral–spatial information, but that information is sensitive to biological variation, environmental conditions, instrument behavior, and analytical design. For food-safety applications, the goal is not to optimize a single metric or to select the most complex algorithm; it is to build models that continue to perform reliably as matrices, sampling conditions, and operating environments change.

## 4. Evidence Across Fungal and Mycotoxin Applications

### 4.1. Early Fungal Infection and Mold-Status Detection

The synthesis below is stratified by analytical endpoint rather than aggregated across all studies. Fungal-status detection, direct or indirect toxin concentration prediction, threshold classification, co-contamination classification, single-kernel sorting, bulk-lot screening, HSI/MSI imaging, and contextual non-imaging NIR evidence are interpreted separately because they answer different analytical questions and support different decisions. This task-level structure is used together with Table 3, Table 5 and Table 6 and Supplementary Table S1 so that the reader can trace how each retained study contributes to the overall evidence map.

Early fungal detection differs from mycotoxin determination. It seeks fungal growth, high spore burden, mold-associated volatiles, or early biochemical change before mycotoxins reach regulatory concern, allowing intervention during grain reception, storage, drying, or sorting.

Yang et al. [11] used VIS–NIR HSI and fungal spore counts to define four mold-status classes in stored maize. Their SAE-CNN-SVM combined spectral and image features and achieved a reported test recognition rate of 98.94%, with sensitivity and specificity of 0.95–1.00. It addressed mold status before direct toxin quantification and visualized results at pixel and kernel levels, but performance came from study-specific training and test sets rather than cross-site external validation.

Siripatrawan and Makino [12] used machine learning-assisted HSI to classify fungal contamination in bulk brown rice. Penicillium-infected grains were mixed with healthy rice at five proportions, and Gaussian SVM achieved 93.4% accuracy from 400 to 1000 nm spectra. GC–MS detected fungal-associated volatiles, supporting the spectral differences. Because the target was contamination proportion rather than measured toxin concentration, the method is an early risk indicator, not a toxin-confirmatory test.

### 4.2. Aflatoxin Risk Assessment in Cereals and Nuts

Maize is the most studied matrix for AI-enabled aflatoxin screening because it is widely consumed, susceptible to Aspergillus, and highly heterogeneous within lots. Kim et al. [10] compared fluorescence, VIS–NIR, SWIR, and Raman HSI in 122 naturally contaminated ground-maize regulatory samples with HPLC aflatoxin values. At a study-specific 10 µg/kg cutoff, fluorescence-QSVM and SWIR-LSVM each achieved 95.7% accuracy with no false negatives in that dataset. The use of natural samples and HPLC reference data strengthens the study.

Zhou et al. [13] classified five aflatoxin B_1_ (AFB_1_) concentration groups in single maize kernels using 1100–2000 nm NIR-HSI. A five-wavelength LDA model reached 95.56% overall accuracy, F1-score 0.9554, and Kappa 0.9444, but independent-test accuracy fell to 88.67%, underscoring the difference between internal results and independent validation.

Bailly et al. [14] analyzed more than 500 maize samples collected across three harvest years using HPLC–MS. Only 7% were contaminated, reflecting real surveillance imbalance. NIR was insufficiently precise for absolute quantification but classified samples above or below European thresholds with 97.4% accuracy for AFB_1_ and 100% for total aflatoxins, suggesting that threshold screening may be more practical than precise regression at very low concentrations.

Kabir et al. [15] applied a lightweight 3D Inception-ResNet to AFB_1_ classification in almonds and reported 90.81% validation accuracy, F1-score 0.899, and AUC 0.964. This more moderate result is informative because almond morphology, thickness, surface variation, and composition complicate spectral classification.

### 4.3. DON, ZEN, Fumonisins, OTA, and Co-Contamination

Evidence for DON, ZEN, fumonisins, OTA, and multi-mycotoxin scenarios is less mature than for aflatoxin. Their molecular structure, concentration range, distribution, fungal ecology, and relation to visible damage differ, so a model for AFB_1_ cannot be assumed to transfer to Fusarium-derived toxins or OTA.

Alisaac et al. [16] studied wheat kernels and flour inoculated with F. graminearum, F. culmorum, and F. poae using VIS–NIR and SWIR HSI, qPCR fungal-DNA measurements, and LC–MS/MS mycotoxin analysis. Reflectance correlated with fungal DNA and DON, particularly in kernels, linking optical data to both pathogen burden and chemical contamination rather than visual classification alone.

Nadimi et al. [17] used NIR-HSI for Fusarium-damaged kernels and DON in Canadian Western Red Spring wheat. With 480 images, 3NN models achieved 85% accuracy and 92% sensitivity for Fusarium damage but 80% accuracy and 77% sensitivity for DON. The lower DON results show the difficulty of inferring toxin presence from indirect spectral signatures; Fusarium damage and DON do not follow a simple linear relation.

Deng et al. [4] addressed a major transferability problem for ZEN and AFB_1_ prediction using FT-NIR and portable NIR datasets. Target-domain correction improved predictions, including R^2^ values of 0.9356 for wheat ZEN and 0.9419 for peanut AFB_1_ with RPD values above 3.9. The evidence concerns adaptation across grain-toxin tasks, not a fully validated source-to-target cross-device protocol.

OTA evidence remains limited. Zhang et al. [18] combined HSI with interpretable deep learning to assess ZEN and OTA in corn grits, relying mainly on artificially contaminated material and a small naturally contaminated validation subset. It provides early wavelength-level, OTA-oriented evidence but not proof of deployment readiness. No patulin-specific food-matrix AI-HSI primary study met the eligibility criteria, so patulin remains an evidence gap.

Most AI-enabled HSI studies target one fungal species or toxin, simplifying model development but not reflecting food-chain conditions in which commodities may carry multiple fungi and mycotoxins. In maize, Aspergillus-derived aflatoxins and Fusarium-derived fumonisins may co-occur.

Kim et al. [7] classified naturally contaminated ground maize as aflatoxin-contaminated, fumonisin-contaminated, co-contaminated, or below cut-off values, using HPLC for aflatoxins and LC–MS/MS for fumonisins. SWIR-HSI with RBF-SVM reached 95.7% accuracy, exceeding fluorescence and VIS–NIR methods, and addressed a multi-hazard decision closer to routine food-chain conditions.

Co-contaminant models are harder because classes increase, prevalence may be unbalanced, and spectral responses can overlap. A classifier may detect fungal-related matrix changes without identifying the responsible toxin; future studies should determine whether models distinguish toxin profiles or only a general contamination-associated state.

Table 5 is an illustrative, stratified evidence matrix rather than an exhaustive ranking or a list of the strongest-performing studies. The nine examples were selected from the complete 20-study evidence map in Supplementary Table S1 by a pre-specified representational logic: at least one example was retained for fungal-status/DON evidence, naturally contaminated aflatoxin screening, co-contamination classification, single-kernel screening, contextual transferability, and early OTA-oriented evidence. Selection was also guided by variation in matrix, contamination origin, reference anchor, measurement unit, sensing modality, validation design, and translational maturity. Studies not included among the illustrative entries in Table 5 remain part of the corpus-level appraisal and are coded in Supplementary Table S1 under the same criteria.

### 4.4. Decision Granularity: Single Kernels, Bulk Samples, and Multimodal Systems

Single-kernel and bulk-sample measurements serve different decisions. Single-kernel analysis supports sorting by removing contaminated units before blending, milling, or processing and may reduce the toxin burden of a batch without discarding all material. This is relevant because aflatoxin and fumonisin contamination can be highly heterogeneous across maize kernels.

Neither approach is inherently superior: single-kernel sensing suits high-throughput sorting and removal, whereas bulk analysis suits lot acceptance, regulatory verification, and legal decisions. A practical system may combine in-line kernel segregation with representative bulk sampling and confirmatory analysis.

Qualitative models classify samples as safe/suspect, contaminated/non-contaminated, or low/moderate/high risk. Accuracy, sensitivity, specificity, precision, recall, F1-score, AUC, and confusion matrices are most useful when linked to an action threshold: accept, reject, segregate, or refer for confirmation.

Strong global regression does not guarantee reliable decisions near a regulatory threshold. Bailly et al. [14] found NIR insufficiently precise for toxin quantification but highly effective for regulatory classification. A method can therefore be useful for screening even if it cannot replace chemical quantification.

Threshold classification and exact quantification are different tasks. Early SWIR-HSI work showed AFB_1_-associated kernel differences, but experimental design, reference-unit alignment, and contamination mechanism determine whether the findings can support real food-safety deployment [19].

No optical modality is universally optimal. Fluorescence can be sensitive to fungal or toxin-associated fluorophores; VIS–NIR captures surface appearance and moisture; SWIR provides bond-related information; and Raman offers molecularly specific signatures. Multimodal systems seek to combine these strengths.

In naturally contaminated ground maize, Kim et al. [10] found the best aflatoxin-screening performance with SWIR and fluorescence models, indicating that sensor selection should follow the contamination target and food matrix rather than technological preference.

Fusion can occur at pixel, feature, or decision level. In Yao et al.’s dual-wavelength fluorescence system, decision-level fusion improved quantitative AFB_1_ prediction over individual wavelength models, suggesting that multimodal or multi-excitation approaches can offset interference and heterogeneous contamination [3].

Full-spectrum HSI can identify wavelengths for reduced-band multispectral systems. In single-kernel almonds, HSI-derived bands enabled a higher-throughput prototype, but natural-contamination validation remained necessary before routine use could be inferred [20].

Multimodal sensing is not an automatic solution. Extra sensors increase calibration complexity, cost, data-management demands, and overfitting risk; fusion merits use only when its advantage persists across batches, instruments, operators, and realistic processing conditions.

### 4.5. Current Challenges in AI-HSI Mycotoxin Translation

A second challenge is translation from model output to action. Food companies need thresholds, reject-actuation reliability, operator protocols, traceability records, confirmatory workflows, and maintenance plans. A model with high internal accuracy may still be unsuitable if it cannot define indeterminate results, preserve audit trails, or trigger appropriate confirmation. The F-AIDRF is proposed to organize these challenges into an explicit checklist for future reporting and industrial evaluation.

Current challenges include limited access to naturally contaminated multicenter datasets, incomplete reporting of sampling and reference-method uncertainty, artificial or visually distinct contamination designs, data leakage through pixel-level or augmented splits, weak reporting of confidence intervals and calibration, poor distinction between fungal status and toxin concentration, and sparse pilot-line evidence. These limitations explain why promising models often remain at proof-of-concept or analytical-screening stages.

The field faces a persistent evidence-to-use gap. Many studies demonstrate that spectral signals can separate prepared classes, but fewer show that the complete sensing-model-decision pipeline remains reliable with naturally contaminated commodities, different seasons, new instruments, realistic prevalence, and moving products. This gap matters for food-industry adoption because screening systems are purchased, maintained, audited, and acted on as operational tools rather than isolated algorithms.

## 5. Safety-Critical Model Evaluation

### 5.1. Metrics, Thresholds, and Error Consequences

AI-enabled HSI is often described by high accuracy or strong regression coefficients, but these alone do not establish safety or fitness for food-chain decisions. Errors are asymmetric: false negatives may release contaminated material, whereas false positives cause avoidable rejection and loss. Evaluation must therefore consider error consequences, the action threshold, prevalence, and reference-method reliability.

Overall accuracy is the proportion of correctly classified observations, but it can be misleading when contamination is rare, or classes are imbalanced. A model applied to a lot containing 95% safe and 5% contaminated products could report 95% accuracy by calling every item safe, yet detect no contaminated sample.

This is especially problematic in mycotoxin surveillance, where positives may be uncommon but costly to miss. Bailly et al. [14] reported high classification performance for naturally contaminated maize but noted that their balanced analytical dataset did not reflect field prevalence. Models trained or tested on balanced classes may therefore look more reliable than they are in routine monitoring.

Accuracy can also mask class-specific failure. In multiclass applications, such as aflatoxin-, fumonisin-, co-contaminated, and below-threshold maize, the majority class may dominate the score. Confusion matrices show which contamination categories are mistaken for one another [7].

Study design also matters. A model may reach 99% accuracy with controlled, visibly distinct, or artificially contaminated samples but fail with natural contamination, different cultivars, altered storage, or samples near the threshold. High accuracy is an early analytical result, not proof of deployment readiness.

Sensitivity (recall) is the proportion of contaminated samples detected, specificity the proportion of safe samples correctly recognized, precision the proportion of predicted positives that are true positives, and F1-score a summary of precision and recall.

Williams et al. [6] showed that accuracy alone is insufficient for imbalanced hyperspectral data and linked high recall to fewer false-negative pistachio classifications. False positives can also create avoidable waste, so evaluation must weigh consumer protection against economic loss.

For quantitative prediction, R^2^ should be considered with RMSEP, RPD, bias, sample distribution, and concentration range. A high R2 may simply reflect wide separation between low- and high-contamination samples while performance near an action threshold remains poor. Zhang et al. [21] therefore reported RMSEP, R^2^p, and RPD together for maize AFB_1_ regression.

F1-score or AUC cannot replace false-negative counts. A model may achieve a strong F1-score while missing an unacceptable number of highly contaminated samples. Safety-related classification requires the full confusion matrix, class-specific sensitivity and specificity, false-negative rate, and threshold-level error distribution.

In food safety, a false negative means that a potentially contaminated item or batch is judged acceptable and may be processed, blended, exported, sold, or consumed. False positives also matter because they trigger rejection, extra testing, diversion, or waste, but the risks are not equivalent.

The operating threshold of an AI-enabled HSI system must match its intended action. A primary-screening model need not make the same determination as confirmatory LC–MS/MS or HPLC; it should identify material for further analysis or segregation. When false-negative control is paramount, a conservative screening threshold may be appropriate.

Bailly et al. [14] distinguished the consumer-safety risk of accepting contaminated maize from the financial cost of rejecting safe material. This distinction should guide threshold selection.

Borderline mycotoxin decisions are affected by reference uncertainty, sample heterogeneity, spectral noise, and model uncertainty. For rapid screening, the operating threshold may need to be more conservative than the final regulatory threshold so doubtful samples enter risk-based confirmation.

Kim et al. [10] illustrate threshold-focused assessment: fluorescence and SWIR models achieved 95.7% accuracy with no false negatives at a 10 µg/kg aflatoxin cutoff in naturally contaminated ground maize. Reporting errors at the selected threshold is more useful for implementation than accuracy alone.

No single metric establishes the safety of an AI-enabled decision. In addition to accuracy, sensitivity, specificity, and false-negative counts, deployment studies should report positive predictive value (PPV) and negative predictive value (NPV) at plausible field prevalence, precision-recall behavior, confidence intervals, threshold and probability calibration, decision-curve or cost-sensitive analysis where appropriate, and explicit rules for indeterminate predictions. Table 7 summarizes complementary measures for release, hold, sorting, and confirmatory testing.

### 5.2. Prevalence, Contamination Design, and Validation Integrity

Class imbalance occurs when safe and contaminated samples are not similarly represented. In routine monitoring, contaminated material may be rare, especially in controlled supply chains, allowing the majority class to inflate apparent accuracy.

Balanced datasets help development by preventing models from predicting only the most frequent class, but balanced test sets alone do not establish field performance. Final models should also be tested at realistic prevalence; Bailly et al. [14] noted that their class-balanced set did not fully represent routine contamination patterns.

Report class-specific recall, precision, false-negative rate, and false-positive rate for each condition. Models should also be challenged with samples near the action threshold, where classification is hardest and most consequential.

Class imbalance is even more problematic in multiclass systems, where toxicologically important co-contamination classes may be rare. In maize, aflatoxin, fumonisin, and co-contamination require separate performance reporting rather than a pooled accuracy score [7].

For this reason, Supplementary Table S1 now codes validation design and leakage-control status for each primary study. The coding distinguishes internal cross-validation or random holdout designs from independent batch, natural-sample, external, transfer, or moving-product evidence. Where preprocessing order, sample grouping, augmentation, or sample-level splitting was not explicit, leakage control was coded as unclear rather than assumed adequate. This links the general methodological concern directly to the retained studies.

Artificial contamination supports method development through controlled concentration ranges, replication, and spectral comparison, but it may not reproduce the biological, chemical, and spatial complexity of natural contamination.

This limitation is well recognized in aflatoxin work. Applied toxin solutions and deliberate fungal inoculation may demonstrate feasibility, but their spectra can differ from naturally contaminated kernels; Wang et al. [8] similarly noted that manual application or laboratory inoculation in peanuts does not mimic natural toxin distribution.

Artificial and inoculated samples remain informative for spectral mechanisms and method development, but should not be treated as equivalent to externally validated natural-contamination studies.

Internal validation includes random train-test splitting, k-fold and repeated cross-validation, and bootstrapping. These methods help optimization but may overestimate performance when observations from the same batch, imaging session, lot, or kernel enter both training and test sets.

In HSI, one food item can yield many spectra, pixels, regions of interest, or repeat images. Splitting related observations across training and test sets lets a model learn item-specific patterns rather than general contamination features. Partitioning should occur at the independent biological sample, batch, lot, or production-event level before augmentation, pixel extraction, or feature engineering.

External validation should use material excluded from all model development and should preferably vary by batch, harvest year, cultivar, storage, contamination level, origin, operator, or instrument. Temporal validation should hold out an entire season or period, whereas cross-batch validation should use complete lots or batches that were excluded from model development.

Instrument transfer remains difficult. Deng et al. [4] documented weak direct transfer across grain-toxin tasks using FT-NIR and portable NIR datasets, but did not complete a source-to-target cross-device deployment study. Internal validation, cross-task adaptation, and true hardware transfer must therefore be distinguished.

Many papers report strong internal validation without enough detail on sample-level partitioning, preprocessing before splitting, hyperparameter selection, or data augmentation. Inglis et al. [2] identified incomplete hyperparameter reporting and limited data or code access as recurring problems, restricting reproducibility and comparability.

Model reliability also depends on training and validation labels. HPLC, LC–MS/MS, ELISA, immunoaffinity methods, fungal DNA quantification, and microbiological methods can provide useful ground truth, but each carries uncertainty from sampling, extraction, calibration, detection limits, recovery, and analytical error.

The imaging unit should match the reference-analysis unit. An individual-kernel model should, where possible, use individual-kernel values or carefully designed pooled measurements; assigning one bulk HPLC result to every pixel or kernel can introduce major label uncertainty when contamination is localized.

Semi-quantitative kits require caution. A portable VIS–NIR corn study used an immunochromatographic assay to define contamination categories and described the device as a screening tool rather than a substitute for precise HPLC quantification. Models trained on categorical kit results should not be presented as direct quantitative toxin-analysis methods.

## 6. Explainable AI and Expert Review

### 6.1. Interpretable Features, Mechanistic Plausibility, and Explanation Quality

This review distinguishes mechanistic explainability from operational auditability. Mechanistic explainability asks whether wavelengths, spatial regions, or features are scientifically plausible indicators of fungal or toxin-associated change. Auditability asks whether the decision can be reconstructed after use through input records, model version, calibration status, threshold, uncertainty flag, operator action, and confirmatory outcome. Post hoc XAI tools may be useful but are not automatically faithful; auditability is mandatory for high-stakes use, whereas post hoc mechanistic explanation is desirable and use-case dependent.

Within F-AIDRF, auditability is treated as a mandatory governance requirement for any high-stakes screen-sort-confirm use, whereas post hoc mechanistic explanations are use-case dependent. A system can be auditable through input traceability, calibration records, model versioning, uncertainty flags, decision thresholds, operator actions, and confirmatory follow-up even when SHAP, LIME, saliency maps, or attention weights are not sufficiently stable to support mechanistic claims. Conversely, a visually attractive explanation does not compensate for a missing audit trail.

Explainable AI clarifies the relation between model inputs and outputs. In HSI food-safety applications, it identifies wavelengths, spatial regions, textures, or feature combinations associated with predicted fungal or mycotoxin risk. SHAP assigns feature contributions to individual predictions, whereas LIME approximates a model locally with an interpretable surrogate [24,25].

This scrutiny matters because HSI often detects indirect signals rather than a mycotoxin molecule. A model may respond to moisture, starch organization, protein denaturation, fungal biomass, roughness, lipid oxidation, or fluorescence interference. Correlation with contamination does not establish a chemical or biological mechanism.

Explainability is not proof of causality. An influential wavelength may reflect a toxin-associated mechanism or a correlated factor such as moisture, particle size, growth stage, illumination, or sample presentation. It strengthens scrutiny but does not replace reference analysis, natural-sample validation, or controlled challenge testing.

Feature selection is an initial interpretability step. Competitive adaptive reweighted sampling, successive projections algorithms, uninformative variable elimination, variable importance in projection, and variable combination population analysis can identify smaller sets of influential bands, reduce dimensionality, improve efficiency, and support translation from laboratory HSI to lower-cost multispectral systems.

Feature selection is not explanation. A band may improve prediction because of a nuisance variable or batch-specific correlation; its relevance requires resampling, external data, mechanistic interpretation, and comparison with reference analysis.

SHAP assigns signed contribution values to each input for each prediction: positive values increase the predicted toxin concentration or contamination probability, and negative values decrease it. Contributions can be aggregated into global importance rankings. Lundberg and Lee [24] introduced SHAP as a unified feature-attribution framework for complex models.

Saliency maps, integrated gradients, and Grad-CAM are useful for spatial–spectral CNNs because they visualize kernel, nut, or food-surface regions that influence a prediction. Grad-CAM uses gradients to localize important image regions [26], while integrated gradients attribute importance to input features in differentiable networks [27].

Attention mechanisms can improve prediction by weighting channels or regions, but attention weights alone are not validated explanations. They require perturbation or ablation tests, agreement with independent attribution methods, and stability across relevant data shifts; otherwise, attention visualizations remain exploratory [28,29,30].

Defensible interpretation links influential wavelengths to plausible matrix changes, such as fungal colonization or toxin-associated effects on starch, proteins, lipids, moisture distribution, hydrogen bonding, or light scattering.

Selected wavelengths generally reflect matrix responses associated with contamination, not unique toxin signatures. Trace-level mycotoxins may not produce isolated peaks in complex foods; direct toxin-detection claims therefore need orthogonal chemical evidence.

Pixel-level pseudo-color maps can visualize possible heterogeneity, but their credibility depends on the training unit. Wang et al. [8] noted that a peanut model trained on average kernel spectra could produce pixel-level maps even though it lacked pixel-level reference labels. Such maps should be read as model-derived risk visualizations, not chemical evidence of toxin location.

A model may achieve excellent accuracy, F1-score, AUC, or regression performance while relying on unstable, non-causal, or dataset-specific signals. A slightly less accurate model may be more transparent, efficient, and suitable for industrial screening; the highest predictive score is not always the best choice.

Assess explanations for faithfulness, not visual appeal. A plausible heat map does not demonstrate that a model relied on meaningful contamination-related signals. Adebayo et al. [30] found that some saliency methods can remain visually persuasive despite weak dependence on model parameters or the data-generating process.

Explainability need not make a model simple; it should make complexity auditable by showing that predictions rest on reproducible, physically meaningful, safety-relevant information.

Explainability belongs in validation, not only presentation. Table 8 summarizes evidence for credible explanation and auditability.

### 6.2. Human Oversight and Auditable Decisions

AI-enabled HSI should support, not replace, expert food-safety judgment. In a screen–sort–confirm workflow, low-risk material may proceed routinely, while high-risk or uncertain material is segregated and referred for confirmation.

Reviewers should assess chemical plausibility of influential wavelengths, proximity to critical thresholds, validity of acquisition conditions, and whether the model remains within its validated domain. New cultivars, unusual moisture, altered packaging or lighting, new sensors, and unfamiliar processing conditions warrant caution.

Oversight is especially important when laboratory models are simplified for deployment. Decisions from compact or reduced-band systems should retain records of selected wavelengths, calibration status, model version, confidence estimate, and follow-up action; missing records should trigger quality review rather than automated action.

Explainable AI requires documented governance covering calibration checks, model-version control, periodic retraining, drift monitoring, uncertainty thresholds, operator training, and confirmatory-testing rules. Final decisions should be traceable to the matrix, reference method, acquisition conditions, model version, explanation output, and reviewer.

Explainability links analytical chemistry, food microbiology, machine learning, and regulatory decision-making, turning opaque prediction into auditable food-safety support.

## 7. Transferability, Domain Shift, and Model Lifecycle Management

### 7.1. Domain Shift and Calibration Transfer

The transferability conclusions in this section are separated by evidence type. HSI and multispectral studies support imaging-specific statements about spatial–spectral models, sample presentation, and sorting relevance. Deng et al. [4] and other non-imaging NIR papers are used only as contextual evidence for calibration transfer, portability, and target-domain adaptation. Their findings justify caution about transfer but do not demonstrate AI-HSI deployment readiness or spatial sorting performance.

Domain shift occurs when the relation between spectra and the target changes from the development setting. In food HSI, changes in storage humidity can alter moisture features, fungal growth, matrix structure, and the apparent spectral relation to mycotoxin risk. Cultivar, season, commodity, contamination mechanism, sample presentation, and reference labels can also shift the prediction problem.

Deng et al. [4] illustrated these challenges across wheat and peanut matrices, AFB_1_ and ZEN targets, and two NIR instruments. Grain species differ in composition and therefore in absorption and reflectance; peanuts, for example, differ markedly from wheat in protein, fat, and sugar content.

Domain shift is not only hardware-related. Models trained on artificially contaminated material may learn signatures of deposited toxin solution, laboratory growth, or controlled moisture rather than the complexity of natural contamination. This is especially problematic for individual-kernel systems, where mycotoxins are localized and heterogeneous.

Transferability is multidimensional. A model may work across batches on one instrument yet fail across cultivars, regions, or sensors; one external test does not establish universal robustness.

Calibration transfer uses mathematical or procedural methods to make spectra from different instruments or conditions comparable enough to retain model utility. Instrument harmonization is broader, adding reference materials, standard procedures, illumination control, maintenance, and quality assurance. Neither alone establishes transfer across commodities or toxins, which requires target-domain evidence.

Calibration transfer should preserve contamination-relevant information while reducing instrument differences. A corrected model may still fail if the target device differs in spectral range, signal-to-noise ratio, illumination geometry, or wavelength resolution.

Deng et al. [4] used FT-NIR and portable NIR spectrometers, but mainly evaluated transfer tasks within the same platform rather than a full instrument-to-instrument deployment experiment. They identified cross-instrument transfer as unresolved because NIR devices differ in range, resolution, illumination intensity, and other acquisition parameters.

Cross-product or cross-mycotoxin adaptation on one instrument does not prove hardware transferability. True transfer requires independent validation of a source model on spectra from another operational device, preferably under real processing conditions.

A defensible harmonization protocol includes a master device, target devices, standardized reference materials, and a transfer set covering safe, borderline, and contaminated samples. After harmonization, assess threshold-specific sensitivity, specificity, false-negative rate, bias, RMSEP, and out-of-distribution detection, not only accuracy or R^2^.

Table 9 summarizes major domain-shift sources and the lifecycle controls needed to preserve analytical validity after deployment.

**Figure 2:**
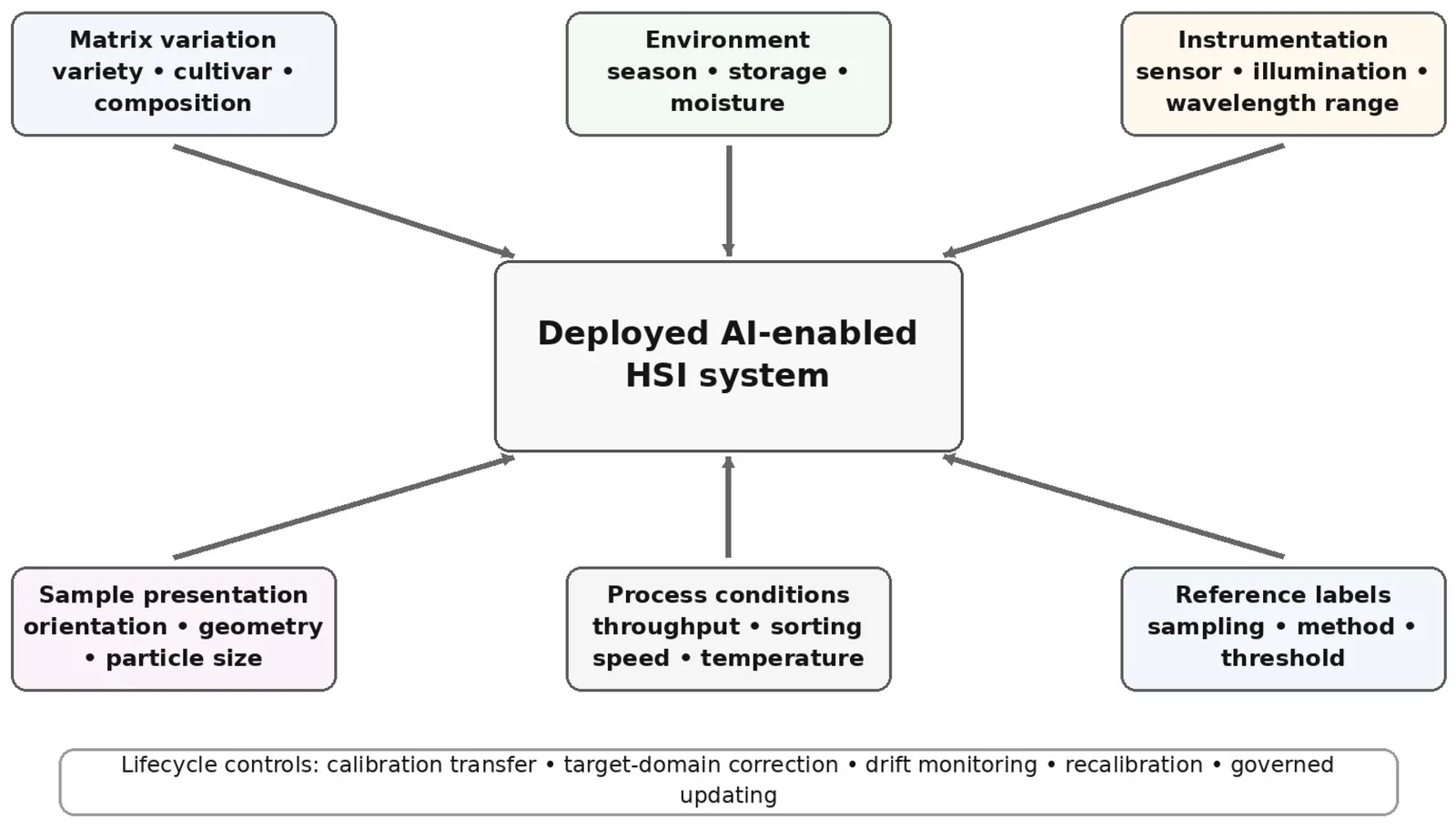
Domain-shift sources and lifecycle controls for deployment-ready AI-enabled hyperspectral systems.

Figure 2 links biological, environmental, instrumental, operational, and reference-label sources of domain shift with lifecycle controls including harmonization, target-domain correction, drift monitoring, recalibration, and governed updating.

### 7.2. Transfer Learning, Model Drift, and Controlled Updating

Transfer learning uses knowledge from a source domain to improve a target domain with limited labels. In food safety, a well-characterized maize or wheat dataset may be transferred to a new cultivar, matrix, toxin, season, or instrument.

Deng et al. [4] showed why direct transfer cannot be assumed: a CNN trained on wheat AFB_1_ performed poorly on peanut AFB_1_ and wheat ZEN targets, with prediction-set R^2^ values of 0.0638, −0.2181, and 0.2167 across direct-transfer tasks.

Retaining the source feature extractor and recalibrating the final regressor with target-domain data improved performance. For FT-NIR, peanut AFB_1_ prediction reached R2 = 0.9419, RMSE = 3.8322 µg/kg, and RPD = 4.1551; wheat ZEN reached R2 = 0.9356 and RPD = 3.9497.

Transfer may reside in general spectral features rather than the complete source model. Target-domain calibration remains necessary because matrices, toxins, and acquisition conditions create task-specific differences.

Target-domain sample size also matters. Deng et al. [4] found that small correction sets produced unstable performance, whereas larger sets improved adaptation and reduced variability. Moderate calibration sets appeared most efficient in their datasets, but the exact size should not be generalized.

Cross-product and cross-mycotoxin transfer requires target-specific chemical reference analysis. Adapted models should be tested with independent naturally contaminated samples, target-relevant ranges, and safety-relevant thresholds, with performance reported separately for each matrix and contaminant.

Deployment begins a model lifecycle rather than ending development. After installation, AI-enabled HSI may drift as the relation between spectral inputs and food-safety outputs changes over time.

Monitor drift with technical and food-safety indicators. Technical measures include spectral distance, control-sample residuals, lamp intensity, detector noise, calibration-panel performance, and wavelength stability; food-safety measures include confirmation disagreement, false negatives, threshold uncertainty, recall changes, and divergence between predictions and reference concentrations.

Automatic retraining after every error is unsafe. Uncontrolled updates can introduce bias, reduce comparability with earlier results, or cause catastrophic forgetting. Zhao et al. [22] identified this risk in cumulative learning, where new data can reduce performance on earlier datasets.

Treat each update as a new analytical version under change control. Records should include a retained historical benchmark, new target-domain samples, independent external validation, threshold-specific false-negative analysis, comparison with the prior model, and approval before replacement.

Transferability is a system property maintained through harmonized measurement, representative target-domain calibration, drift detection, controlled updating, and safety-oriented revalidation. A model is transferable only when it remains reliable after biological, technological, and operational conditions change.

## 8. Deployment-Ready AI-Enabled Hyperspectral Systems

### 8.1. Pilot-Line Translation, Throughput, and Sorting

Operational evidence should be reported quantitatively. At minimum, studies intended for deployment should state units processed per second, conveyor speed, image-acquisition time, end-to-end decision latency, reject-actuation timing and accuracy, product-overlap rate, downtime, calibration frequency, environmental tolerance, computing requirements, confirmation workload, false-rejection cost, and total cost of ownership. Without these values, claims of line-readiness remain qualitative.

Most AI-HSI studies remain laboratory proof-of-concept work, relying on controlled illumination, fixed orientation, homogeneous backgrounds, small datasets, and off-line computing. These conditions demonstrate feasibility but not the variability of commercial handling, sorting, storage, or processing lines.

Almonds illustrate the transition challenge. HSI can detect AFB_1_ non-destructively, but full-spectrum systems are slowed by acquisition and processing demands. A practical route is to identify robust wavelengths with HSI and implement a reduced-band multispectral sorter at industrially compatible speed.

Laboratory systems should be designed for deployment by selecting wavelengths stable across lots, acquiring moving products, testing orientation and overlap, and ensuring the model can run on line-available computing hardware.

Industrial use requires sufficient throughput without loss of analytical validity. Conveyor speed, scan rate, exposure, spatial resolution, spectral range, number of bands, field of view, sample spacing, processing latency, and reject timing all determine performance.

Full HSI produces rich spectral–spatial data but creates substantial computational demands because hundreds of bands require correction, segmentation, processing, and interpretation before sorting. This drives interest in reduced-band multispectral systems after robust research wavelengths are identified.

Individual-kernel and nut sorting requires edge processing before product reaches the reject point. Acquisition hardware may reduce raw HSI to compact features, selected bands, or embedded-model outputs while retaining quality-control records, decisions, and exceptions needed for audit and revalidation.

Portable systems can provide rapid on-site screening but are not necessarily industrial high-throughput tools. Narrow coverage, simplified labels, or limited validation may restrict generalisability; until independent evidence supports the use case, they should be presented as preliminary screening tools.

Individual-kernel screening is valuable for heterogeneous contamination because a few highly contaminated kernels can determine lot safety. Removing them rapidly can reduce toxin burden without rejecting the whole batch.

Single-kernel optical screening can identify high-risk kernels directly, reducing bulk-sampling skewness, and can remove them before milling, blending, storage, or processing.

The reject mechanism is as important as the classifier. Even a highly accurate model has little value if signals are late, kernels overlap, conveyor speed fluctuates, or rejected material remains mixed with accepted material. Pilot-line studies should report mechanical sorting efficiency alongside analytical performance.

Stationary-kernel studies aid spectral characterization but cannot establish high-speed sorting suitability. Moving-kernel analysis provides stronger evidence of industrial feasibility.

### 8.2. Traceability, HACCP Integration, and Governance

A worked use case illustrates the intended role. At grain reception, an AI-HSI unit could screen incoming maize, flag high-risk kernels or sublots, and assign each sample to accept, hold/resample, or segregate/confirm. Lots with low-risk optical profiles would continue under routine verification; uncertain lots would be held for intensified sampling; high-risk lots would be segregated and submitted to HPLC or LC–MS/MS. The system would not issue a final legal mycotoxin determination; it would create a faster triage layer with auditable links to confirmation.

Sensor fusion may improve robustness when one modality is insufficient: fluorescence can respond to fungal metabolites or endogenous fluorophores, while VIS–NIR and SWIR capture surface condition, moisture, composition, and structure. It also increases calibration, data-management, and maintenance demands.

For deployment, fusion should demonstrate a safety-critical advantage, particularly fewer false negatives near action thresholds, rather than a small accuracy gain alone; the architecture must remain feasible at line speed.

Each inspection record should capture lot or supplier identifier; date, time, and line; product and matrix; sensor and illumination settings; model and calibration version; confidence score; decision class; operator action; sorting outcome; and any confirmatory result.

These records support risk-based supplier monitoring, targeted sampling, trend analysis, and preventive action, and can reveal changes in prevalence, moisture, fungal pressure, or model performance over time.

Data integration creates governance duties. Networked or cloud-connected systems need access control, backups, audit logs, cybersecurity safeguards, and protection against unauthorized model changes. Although rarely described in AI-HSI papers, these controls are necessary in industry.

AI-HSI should be embedded in HACCP-based food-safety management, not used as a stand-alone demonstration. Its role depends on food-chain stage, intended decision, and commodity-specific hazard analysis.

Within HACCP systems, HSI may serve in prerequisite programs, operational prerequisite programs, or critical control procedures, depending on product, process, hazard analysis, and regulation. A contamination prediction alone does not create a critical control point.

At maize reception, HSI could classify lots as low risk, uncertain, or high risk: low-risk lots proceed under routine verification, uncertain lots are held for more sampling, and high-risk lots are segregated for HPLC or LC–MS/MS confirmation. This combines rapid triage with the legal defensibility of reference analysis.

Naturally contaminated maize studies at regulatory-relevant cutoffs support this role. Kim et al. [10] concluded that HSI screening could reduce the time and labor of routine confirmatory workflows while retaining chemical confirmation for final decisions.

Deployment cost extends beyond the camera to illumination, conveyors, singulation, computing, calibration standards, maintenance, validation, software, training, laboratory-information-system integration, and periodic confirmation.

Costs may fall when full HSI is translated into reduced-band multispectral systems after robust wavelength selection, especially for almonds, peanuts, maize, and pistachios where selected bands may support rapid in-line sorting.

Training should cover routine use, invalid measurements, calibration failure, product overlap, abnormal confidence scores, and operation outside the validated domain, including when to escalate to quality-assurance staff.

Regulatory acceptance depends on intended use. At present, AI-HSI is most immediately suited to four functions:

Rapid screening of incoming lots at reception;

Sorting of individual kernels or nuts;

Prioritization of samples for confirmatory analysis; and;

Early warning during storage and processing.

AI-HSI is unlikely to replace HPLC, LC–MS/MS, or validated immunochemical procedures for final legal mycotoxin determinations. Reference methods provide traceability, defined detection limits, validation parameters, and legal defensibility.

The sound deployment model is AI alongside reference analysis. Optical systems can reduce the number of samples needing costly chemical confirmation, speed intervention, and support targeted sorting, while reference methods remain essential for calibration, validation, regulatory verification, dispute investigation, and lifecycle monitoring.

A system is deployment-ready only when it supports a reliable pathway for risk detection, sorting or holding suspect material, preserving digital evidence, triggering confirmation, and showing that routine decisions remain protective.

### 8.3. Regulatory Context, Decision Thresholds, and Confirmatory Analysis

Regulatory and screening thresholds are not interchangeable. This review now treats EU, Codex, and FDA materials as contextual regulatory or standards/guidance sources. EU Regulation 2023/915 sets commodity-specific maximum levels for selected contaminants, and EU Implementing Regulation 2023/2782 specifies sampling and analytical requirements for official mycotoxin control [31,32]. Codex CXS 193-1995 provides international maximum levels and sampling-plan guidance for traded commodities [33], while FDA CPG Sec. 555.400 provides nonbinding enforcement guidance for aflatoxins in human food [34]. None establishes AI-HSI as a substitute for official reference analysis. Studies should state jurisdiction, commodity category, analyte, sampling unit, reference method, and the rationale for the action threshold. The 10 µg/kg ground-maize cutoff is matrix- and design-specific, not a universal legal limit.

### 8.4. Complementary Optical Technologies and the Boundary of This Review

The evidence map centers on HSI and related spectral imaging because spatially resolved measurements support the proposed screen–sort–confirm applications. FT-NIR, portable NIR, handheld Raman, electronic-nose, and smartphone tools may complement HSI, but their sensor physics, data units, and deployment evidence differ and were not assessed comprehensively; they are not interchangeable with AI-HSI evidence. Recent reviews were used as contextual sources to situate these boundaries within broader non-destructive food-safety sensing [35,36].

F-AIDRF may be adaptable to these technologies because its six domains address evidence quality rather than one sensor, but each needs technology-specific criteria for sampling, reference alignment, calibration transfer, uncertainty, throughput, and workflow. Deng et al. [4] show that portability alone does not establish transferability or deployment readiness.

## 9. Food–AI Deployment-Readiness Framework

### 9.1. Framework Development, Intended Users, and Limits

F-AIDRF arose from recurrent gaps identified in the corpus: weak unit alignment, incomplete reference-quality reporting, reliance on artificial contamination, limited external validation, incomplete false-negative analysis, weak explainability or auditability, little transfer testing, sparse operational evidence, and unclear links between model output and protective food-industry action. These gaps were grouped into six domains through interpretive synthesis rather than statistical derivation. The six domains correspond to the main failure points observed when a laboratory optical model is translated toward food-industry use: analytical validity, algorithmic robustness, explainability and auditability, transferability and maintenance, operational feasibility, and decision integration.

The derivation was deliberately conservative. Analytical validity was derived from repeated unit-mismatch and reference-label concerns; algorithmic robustness from class imbalance, leakage, and threshold-error concerns; explainability and auditability from the need to inspect and reconstruct AI-assisted decisions; transferability and maintenance from domain-shift and calibration-transfer limitations; operational feasibility from throughput, sorting, and cost constraints; and decision integration from the need to connect outputs to accept, hold, sort, refer, or reject actions. These domains are therefore evidence-derived categories for organizing review findings, not empirically validated scales.

The novelty of F-AIDRF is not the isolated claim that models require validation, explanation, or HACCP-compatible decisions. Its contribution is the non-compensatory integration of these requirements into a food-safety-specific appraisal pathway. Technology-readiness approaches generally emphasize technology maturity; analytical-validation approaches evaluate method performance; and HACCP-based systems organize hazard control. F-AIDRF links these perspectives by asking whether an AI-enabled HSI system has enough evidence to support a defined food-chain decision while preserving confirmatory analysis, auditability, and false-negative control.

F-AIDRF differs from general technology-readiness levels because it is not a maturity ladder for hardware alone; it asks whether an AI-enabled optical system can support a defined food-safety decision. It differs from analytical-method validation because it does not itself validate a method, define official performance criteria, or replace laboratory confirmation. It also differs from HACCP because it does not identify hazards for a particular process; instead, it helps researchers and quality-assurance teams judge whether the evidence behind an AI-HSI tool is sufficient for a proposed screening, sorting, hold, or confirmation-referral role. Independent validation of the framework will require multiple assessors, prospective pilot-line case studies, and comparison with real decision outcomes.

### 9.2. Demonstration of the Framework Within the Evidence Corpus

Supplementary Table S1 applies the framework descriptively to all 20 study entries, comprising 17 spectral-imaging studies and three contextual non-imaging NIR studies. For HSI-equivalent readiness staging, the denominator is the 17 spectral-imaging studies: 13 were classified as R1 and four as R2. The three contextual non-imaging NIR studies are labeled separately and are not included in this HSI-equivalent R1/R2 count; none of the 20 study entries met the proposed R3–R5 conditions. The result describes reporting and translational maturity within this evidence map, not validation of F-AIDRF.

The demonstration also has limits. One author conducted the appraisal, no inter-rater reliability was calculated, and the framework was applied to the same evidence corpus from which its domains were derived. This application should therefore be understood as a worked demonstration of the checklist, not as validation. It shows how the framework exposes missing evidence, but it does not prove that different assessors, industry users, regulators, or technology developers would assign identical readiness stages.

To make the distinction explicit, F-AIDRF contains two components that should be evaluated separately in future work: construct validity, meaning whether the six domains capture the evidence that matters for AI-HSI food-safety decisions; and classification reliability, meaning whether independent assessors assign the same stage to the same technology using the same rules. The present manuscript addresses construct rationale and descriptive use; it does not claim either independent construct validation or classification reliability.

In practice, users define the intended decision and sample unit, map evidence across six domains, identify the first unfulfilled gate, and plan the next validation step. A lot-screening model with chemical ground truth but no external validation may be an R2 candidate for further study, but not for routine sorting or compliance decisions.

### 9.3. Core Evidence Domains

F-AIDRF turns evidence into decision-relevant questions. Its non-compensatory design prevents strong performance in one domain, especially internal accuracy, from masking weak ground truth, false-negative control, transferability, or governance. It identifies minimum evidence for a defined use case; it does not produce a universal score or regulatory decision.

A model trained on bulk HPLC values is not automatically an individual-kernel toxin detector, and visual mold grading is not mycotoxin quantification. Analytical validity requires alignment between the claimed output and ground truth.

Naturally contaminated regulatory maize samples, low cut-offs, and explicit false-negative assessment provide stronger analytical evidence than models based only on visibly distinct or artificially spiked material [10].

Algorithmic robustness is the ability to remain reliable under unseen but relevant variation and depends on credible design as well as reported performance.

Robust models need acceptable recall and false-negative control, especially near regulatory thresholds; contaminated pistachios classified as safe illustrate why recall and false-negative assessment must accompany accuracy.

Within F-AIDRF, high accuracy cannot compensate for missing independent validation, poor class representation, absent false-negative reporting, or likely data leakage.

Explainability and auditability allow predictions to be inspected, reconstructed, and defended after a decision, which is essential when food is classified as acceptable, suspect, or unsuitable.

Explainability should test whether a model relies on credible contamination-related information rather than unstable background, illumination, orientation, or imaging-session effects.

A highly accurate model may still be unsuitable for high-stakes food-safety decisions when it cannot credibly explain its conclusion.

Transferability is the ability to remain reliable when matrices, cultivars, contamination conditions, storage, instruments, or acquisition settings change; maintenance preserves that reliability after deployment.

Deng et al. [4] found that a wheat AFB_1_ source model performed poorly on peanut AFB_1_ and wheat ZEN data, whereas partial target-domain recalibration improved performance. Transferability is therefore an ongoing capability supported by calibration, monitoring, and controlled adaptation, not a fixed model property or evidence of fully validated cross-instrument deployment.

A random holdout from the same experiment does not establish transferability.

Table 10 converts each F-AIDRF domain into a decision question and minimum evidence expectation; Supplementary Table S1 applies the same domains with explicit single-author limitations.

**Figure 3:**
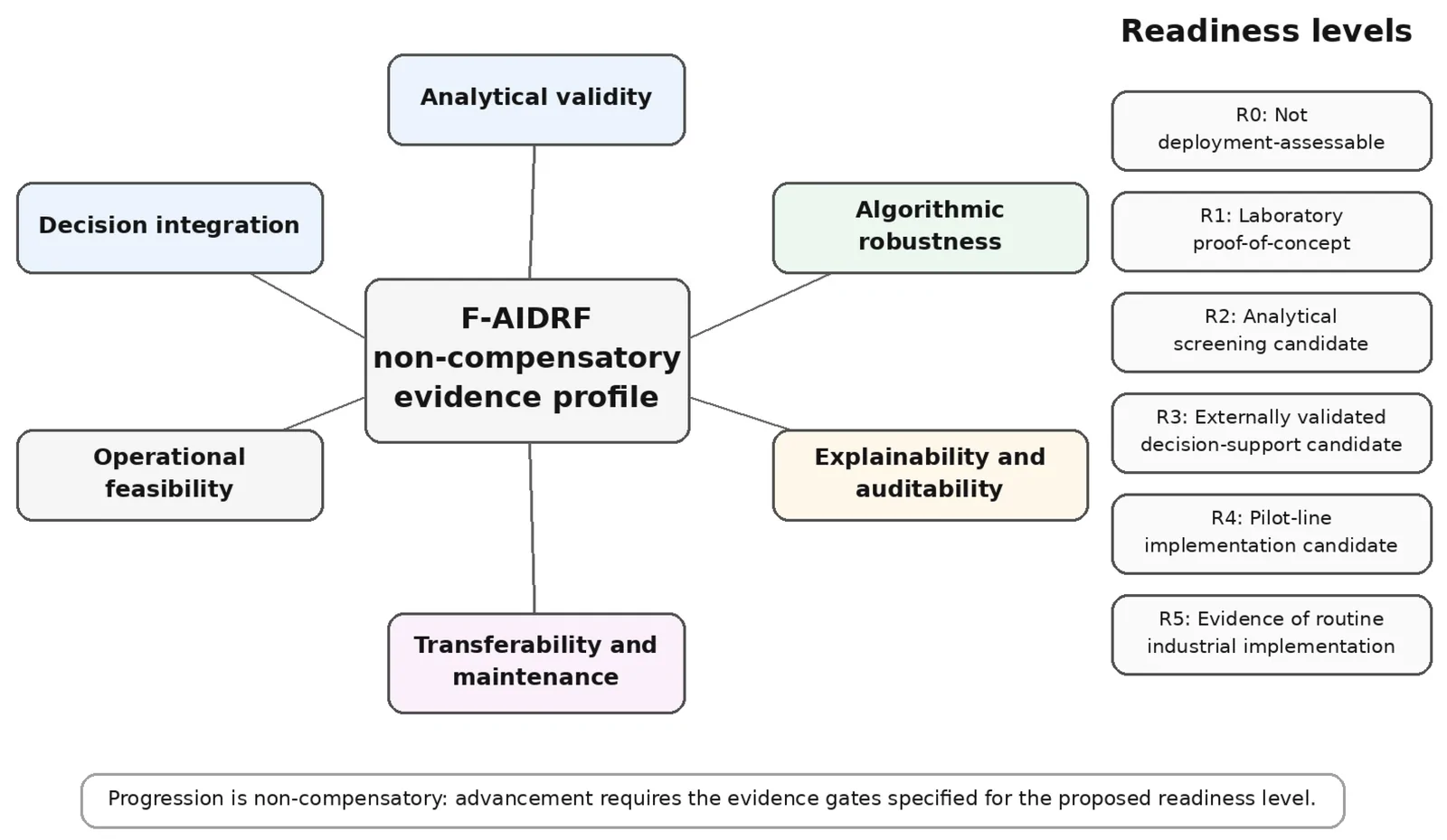
Proposed Food–AI Deployment-Readiness Framework (F-AIDRF) and provisional non-compensatory translation stages.

Figure 3 presents F-AIDRF as a preliminary non-compensatory architecture in which all six domains constrain progression from laboratory feasibility to more demanding operational evidence; it is not a certification ladder.

### 9.4. Operational Integration and Provisional Readiness Gates

Operational feasibility asks whether a system can work under real processing conditions. Laboratory HSI may perform strongly yet remain unsuitable because of slow acquisition, data volume, computational delay, cost, or poor conveyor and sorter integration.

Reduced-band multispectral systems may bridge laboratory HSI and industry. In one almond study, full HSI inspected about 10 almonds per minute and a reduced-band system 150, but artificial contamination and missing natural-sample validation limit deployment claims [20].

Deployment requires an explicit decision pathway: who acts on predictions, how uncertain material is handled, and when confirmation is triggered.

AI-enabled HSI is most suitable for screening, sorting, prioritization, and early warning; final legal or regulatory decisions require validated reference methods. Naturally contaminated maize studies suggest HSI can reduce confirmation time and labor rather than replace chemical analysis [10].

F-AIDRF defines six provisional translation stages without averaging scores. A failure in reference validity, external validation, or confirmation logic blocks progression even when accuracy is high.

Progression requires traceable ground truth beyond R1, independent holdout and threshold-specific error analysis beyond R2, external or transfer evidence beyond R3, and moving-product or pilot-line tests beyond R4. R5 denotes controlled routine implementation with governance and confirmation, not regulatory approval.

A system cannot exceed R3 without evidence of transferability beyond its original dataset.

The profile remains descriptive rather than numerical so that high predictive accuracy cannot hide failures in false-negative control, external validation, or governance.

F-AIDRF organizes interpretation across the evidence domains, but its criteria, readiness boundaries, and utility need prospective testing across matrices, sensors, operating conditions, and independent assessors before broader standardization.

Table 11 stages are descriptive and non-regulatory: they reveal missing evidence but do not certify systems, replace validation, or determine legal compliance.

### 9.5. Food-Industry Applications of F-AIDRF

For food companies, F-AIDRF is intended as a pre-adoption and pilot-design tool. It can help compare supplier claims, identify missing validation evidence, determine whether a system is suitable for screening or sorting rather than confirmation, and define which additional tests are needed before routine use. The framework is most useful when applied to a specific commodity, decision point, action threshold, and workflow rather than to a technology in general.

Table 12 translates the framework into practical industry use cases. These examples do not imply that current systems already satisfy the evidence requirements; they show how the framework can be used to plan validation, procurement, and quality-assurance decisions.

## 10. Limitations of the Review and the Preliminary F-AIDRF

This review has several limitations. It is a critical narrative review with structured evidence appraisal, not a PRISMA-compliant systematic review. The evidence map is a focused corpus rather than an exhaustive census, and the appraisal was conducted by a single author. This single-author design is not merely a procedural detail; it directly affects the confidence that can be placed in readiness-stage assignment. The stages should therefore be interpreted as conservative, transparent descriptors of reported evidence, not as definitive classifications of study quality or technology value.

The F-AIDRF itself is preliminary. The six domains and R0–R5 descriptors were derived from recurring evidence gaps in the retained corpus and were applied to the same corpus. This creates conceptual circularity and prevents the present review from claiming framework validation. The framework is best understood as a hypothesis about which evidence domains should govern AI-HSI translation, together with a practical coding template for future independent testing.

A further limitation is that readiness boundaries remain partly judgment-based because published studies differ in reporting detail. Future work should test the checklist with multiple independent assessors, measure agreement, refine mandatory and desirable criteria, apply the framework to external evidence sets, and evaluate whether higher F-AIDRF stages predict reliable performance in pilot-line or routine industrial settings.

## 11. Future Perspective

### 11.1. Evidence Boundaries, Benchmark Datasets, and Naturally Contaminated Evidence

Future progress should move from proof-of-concept demonstrations toward decision-centered, industry-relevant validation. The key objective is not simply higher model accuracy, but evidence that a defined optical-AI workflow can reduce risk, control false negatives, manage indeterminate outcomes, preserve traceability, and coexist with confirmatory analysis under realistic food-chain conditions.

The primary corpus concentrates on aflatoxin applications, especially maize, nuts, and individual-kernel screening. OTA evidence remains early-stage and no patulin-specific food-matrix AI-HSI study met the criteria. F-AIDRF may be conceptually relevant across hazards, but its empirical basis is strongest for aflatoxins and selected Fusarium targets. Priorities include benchmark datasets with naturally contaminated material, decision-relevant ranges, multi-site acquisition, and documented chemical or biological reference analysis.

### 11.2. Prospective Industrial and Independent Framework Validation

Benchmark datasets are needed to compare platforms, preprocessing, algorithms, and validation strategies. Current studies differ widely in matrix, sample size, contamination origin, range, reference method, class distribution, partitioning, and metrics, so apparent superiority may reflect design rather than analytical advantage.

Benchmarks must prevent leakage: spectra, pixels, or repeated images from one kernel should never appear in both training and test sets. The independent unit is the biological sample, batch, or lot, not the number of pixels from one item.

Well-designed benchmarks would reduce fragmentation and distinguish genuine methodological advances from dataset-specific gains.

Multi-center work also needs a shared reference-analysis protocol. Sampling and imaging may be local, but confirmation should be harmonized through validated HPLC, LC–MS/MS, or equivalent methods to limit inconsistency from extraction, thresholds, laboratories, or calibration standards.

Most studies use retrospective datasets that are collected, reference-tested, and then split into training and test sets. This is appropriate for initial development but does not show whether a system remains reliable after installation.

The strongest prospective design compares AI-HSI screening with a predefined confirmation pathway: low-risk lots continue under routine verification, uncertain lots are held for intensified sampling, and high-risk lots are segregated for HPLC or LC–MS/MS.

F-AIDRF also requires validation through a staged program that separates framework derivation from framework evaluation. First, an external evidence set should be assembled from studies not used in the present review, ideally including at least 30 additional AI-enabled optical food-safety studies across HSI, multispectral imaging, portable spectroscopy, and related screening technologies. Second, two to four independent assessors with expertise in mycotoxin analysis, optical sensing, food-industry quality assurance, and machine learning should be trained using the coding manual in Supplementary Table S3 and asked to code the same studies independently. Third, inter-rater reliability should be quantified for domain-level ratings and R0–R5 stages using percentage agreement, Cohen’s or Fleiss’ kappa for categorical decisions, and intraclass correlation or weighted agreement where ordinal ratings are used. Fourth, disagreements should be resolved through an adjudication meeting, with reasons recorded so that ambiguous rules can be revised.

A second validation layer should use expert elicitation or a Delphi process. Experts from food-industry quality assurance, regulatory laboratories, mycotoxin analytics, hyperspectral engineering, and AI governance should review the six domains, mandatory criteria, and stage boundaries through at least two anonymized rounds until consensus or documented disagreement is reached. A third layer should compare F-AIDRF profiles with prospective pilot-line outcomes, such as false-negative control, indeterminate-rate management, confirmation workload, reject-actuation accuracy, downtime, and cost of ownership. Only after these external, inter-rater, Delphi, and pilot-line tests should F-AIDRF be considered for broader standardization or routine use.

Reduced-band multispectral systems may aid industrial translation. In one single-kernel almond study, laboratory HSI scanned about 10 almonds per minute and a selected-wavelength system about 150; because almonds were artificially contaminated, natural-contamination validation remains necessary [20].

### 11.3. Uncertainty, Affordable Deployment, and Multi-Hazard Platforms

Future food-safety AI should report a prediction with confidence, uncertainty, and model applicability. Results near a regulatory threshold should not be handled like clearly low- or high-risk results.

Safety-oriented systems should abstain from automated accept/reject decisions when uncertainty is high and route these samples through an ‘uncertain’ pathway for additional sampling, repeat imaging, or laboratory confirmation.

The preferred system is an uncertainty-aware decision-support model that explains why a sample was flagged, reports confidence, and indicates whether the sample is within the validated operating domain, not a black box reporting accuracy alone.

Full HSI can be costly and computationally demanding, so it may be unsuitable for some processing environments. Future work should translate laboratory findings into lower-cost multispectral, portable, line-scan, or embedded platforms.

Portable and reduced-band systems may aid grain reception, storage monitoring, farm-level screening, and feed processing, but their smaller spectral information must be weighed against practical advantages and each system must be tested against a traceable reference method at decision-relevant thresholds.

Portable VIS–NIR work in corn shows both opportunity and limitation: rapid classification can support on-site screening, but the device is described as a complement to HPLC quantification [23].

Food systems rarely face one hazard. Commodities may carry multiple fungi, mycotoxins, pesticide residues, heavy metals, moisture-related deterioration, insect damage, or physical defects, so future work should move from single-target detection to multi-hazard assessment.

Fusion is justified only when it improves safety-critical performance, reduces false negatives, strengthens threshold robustness, or improves sorting—not merely because multiple sensors are available.

### 11.4. Open Science and Reporting Standards

AI-HSI studies are difficult to reproduce when preprocessing, hyperparameters, architecture, wavelength selection, calibration, or validation are incompletely reported. Inglis et al. [2] noted frequent gaps in hyperparameter reporting and limited access to open-source code in mycotoxin machine-learning research.

Open data should be encouraged where legal and commercial conditions permit. When full release is restricted by intellectual property, supplier agreements, or commercial limits, options include controlled-access repositories, anonymized spectra, metadata-only releases, benchmark subsets, synthetic data, or federated learning.

Performance claims should state model construction, sample selection, assumptions, validation, and the conditions under which predictions should not be trusted.

## 12. Conclusions

AI-enabled hyperspectral imaging has clear research value for fungal and mycotoxin risk management, but current evidence supports cautious use as a screening, sorting, prioritization, and confirmatory-referral technology rather than routine industrial or regulatory replacement of validated reference methods. The most informative studies align the optical unit with the reference-analysis unit, use natural or industrially realistic contamination, report safety-critical metrics, control data leakage, and test transferability beyond the original dataset.

AI-enabled HSI does not replace HPLC, LC–MS/MS, or other validated reference methods for final regulatory decisions. Optical systems usually infer risk from contamination-associated matrix changes and depend on aligned reference methods, representative sampling, false-negative control, natural-contamination evidence, and validation across changing domains. Its near-term role is screen–sort–confirm: rapid triage, targeted sorting or holds, and representative confirmation when decisions require it.

F-AIDRF is a preliminary, non-regulatory tool for revealing evidence gaps. It is not validated, does not certify technologies, and cannot replace formal method validation, commodity-specific HACCP analysis, or legal compliance assessment. Its purpose is to prevent high accuracy from masking weaknesses in analytical validity, transferability, feasibility, or governance.

Priorities include naturally contaminated multicenter datasets, prospectively defined industrial trials, transparent reporting, uncertainty-aware models, technology-specific cost and workflow analysis, and independent F-AIDRF validation. Until then, AI-enabled HSI should be used as decision support, not as a deployment-ready substitute for validated laboratory analysis.

## Figures and Tables

**Table 1:** Evidence-identification and selection record for the focused critical narrative corpus.

Component	Implementation in This Review	Interpretive Limit
Review type	Critical narrative review with structured evidence appraisal; not PRISMA-compliant and not a pooled systematic review.	Supports transparent critical synthesis and framework development, not exhaustive prevalence estimates.
Databases/sources checked	Web of Science, Scopus, PubMed, ScienceDirect/publisher records, Google Scholar citation chaining, and official EU, Codex, and FDA sources; final checks through 1 July 2026.	Database coverage differed by source; citation chaining was used to reduce, not eliminate, selection bias.
Core query logic	(hyperspectral OR multispectral OR spectral imaging OR fluorescence OR Raman OR VIS–NIR OR SWIR) AND (AI OR machine learning OR deep learning OR chemometrics OR transfer learning OR calibration transfer OR explainable AI) AND (fungal OR Aspergillus OR Fusarium OR Penicillium OR aflatoxin* OR fumonisin* OR DON OR ZEN OR OTA OR patulin OR mycotoxin*) AND (food OR feed OR grain OR maize OR wheat OR peanut OR almond OR pistachio OR rice OR flour).	Exact syntax was adapted to database fields; Supplementary Table S2 lists query blocks and eligibility logic.
Duplicate handling	Records were de-duplicated by DOI, exact or near-exact title, first author, year, and publisher version; duplicates and secondary copies were removed before eligibility coding.	The process is reconstructed for transparency and does not replace a protocol-registered search audit.
Screening record	Seventy-four candidate records were documented during the focused evidence-mapping process; 14 duplicate or secondary records were removed; 60 records were screened by title/abstract/full text. The combined 20-study evidence map consisted of 17 spectral-imaging studies and three contextual non-imaging NIR studies; five reviews, seven methodological papers, and four regulatory/guidance sources were retained for context.	Numbers describe the documented focused corpus only; they should not be read as field-wide retrieval counts.
Exclusion categories	Exclusions included non-food matrices; non-imaging or non-spectral methods outside contextual scope; descriptive spectroscopy without a predictive model; general quality/composition studies without fungal/mycotoxin endpoint; lack of traceable reference target; duplicate reports.	Detailed category-level exclusion rules are provided in Supplementary Table S2.
Primary inclusion	Peer-reviewed spectral-imaging study; AI/ML/chemometric model; food/feed matrix; fungal status or mycotoxin target; traceable reference or validated label.	Evidence strength still depends on reference quality, unit alignment, contamination design, validation integrity, and decision relevance.
Contextual inclusion	Three non-imaging NIR studies retained only for portability, threshold screening, or calibration-transfer context.	Contextual NIR evidence is not treated as HSI or spatial sorting evidence.

**Table 2:** Structured extraction variables used to stratify the evidence map before F-AIDRF staging.

Extraction Variable	How It Was Used in the Synthesis
Endpoint category	Studies were separated as fungal-status detection, toxin concentration prediction, threshold classification, co-contamination classification, or contextual portability/transferability evidence.
Measurement unit	The appraisal recorded whether the model operated at pixel, kernel/nut, powder, bulk-sample, lot, or dataset level.
Reference-analysis unit	The extraction distinguished chemical toxin values, fungal DNA or spore burden, visual damage, contamination proportion, and other validated reference targets.
Contamination design	Natural, inoculated, spiked, mixed, and unclear designs were recorded separately because they support different readiness claims.
Validation design	Internal split, cross-validation, independent holdout, external-domain testing, transfer testing, and line/pilot evidence were coded explicitly.
Operational evidence	Throughput, sorting/hold action, confirmatory referral, calibration, traceability, and governance evidence were recorded when reported.

**Table 3:** Concise study-level readiness summary from the combined 20-study evidence map, including three contextual non-imaging NIR studies. Ctx., contextual non-imaging evidence.

Study	Endpoint/Unit	Stage	Failed Gate	Rationale for Classification
Yao et al. [3]	AFB_1_; single-kernel fluorescence HSI	R1	External/pilot-line evidence	Strong mechanistic and fusion evidence, but controlled inoculation and study-specific validation limit screening-readiness inference.
Williams et al. [6]	Aflatoxin infection; pistachio images	R1	External/pilot-line evidence	Recall is relevant to false-negative control, but deployment, transfer, and line evidence are absent.
Kim et al. [7]	Aflatoxin/fumonisin/co-contamination; ground maize	R2	External decision-support evidence	Natural samples and chemical references support analytical screening, but cross-site and moving-line validation were not reported.
Wang et al. [8]	Aflatoxin risk; peanut HSI	R2	External transfer and line evidence	Natural contamination strengthens relevance, but stage remains limited by study-specific validation and no operational test.
Chu et al. [9]	AFB_1_ classes; maize kernels	R1	Natural/external validation	Kernel-level design is relevant to sorting, but field-inoculated samples and early validation limit readiness.
Kim et al. [10]	Aflatoxins; ground maize	R2	External/pilot-line evidence	Natural regulatory samples and no false negatives at a study-specific cutoff support screening-candidate status only.
Yang et al. [11]	Mold status; stored maize	R1	Toxin/reference alignment	High mold-status recognition does not establish mycotoxin concentration or regulatory risk.
Siripatrawan and Makino [12]	Fungal contamination; bulk rice	R1	Mycotoxin/reference alignment	Classifies contamination proportion rather than toxin concentration; no external deployment.
Zhou et al. [13]	AFB_1_ groups; maize kernels	R1	External robustness	Independent-test drop indicates limited generalization beyond study conditions.
Bailly et al. [14]	Aflatoxin thresholds; ground maize NIR context	Ctx. R2	HSI/spatial evidence	Useful threshold-screening context but not imaging or spatial sorting evidence.
Kabir et al. [15]	AFB_1_; almonds	R1	Natural/external validation	Deep-learning classification remains laboratory-stage; natural and operational validation are insufficient.
Alisaac et al. [16]	Fusarium/DON; wheat kernels/flour	R1	Decision-level validation	Strong biological and chemical reference context, but no deployment or cross-domain decision evidence.
Nadimi et al. [17]	Fusarium damage and DON; wheat	R1	DON sensitivity and deployment	DON detection lagged Fusarium-damage detection; operational use was not demonstrated.
Zhang et al. [18]	ZEN/OTA; corn grits	R1	Natural/external validation	Early OTA-oriented XAI evidence, but mainly artificial contamination and limited natural subset.
Kandpal et al. [19]	AFB_1_; corn kernels	R1	External/pilot-line evidence	Early SWIR feasibility without sufficient operational or external readiness evidence.
Mishra et al. [20]	Aflatoxin; almonds HSI/MSI	R2	Natural validation	Throughput-oriented MSI prototype supports screening development, but artificial samples limit readiness.
Zhang et al. [21]	AFB_1_ quantification; maize	R1	External threshold validation	Regression evidence remains study-specific; line and transfer evidence absent.
Zhao et al. [22]	Aflatoxin pixel-level detection; peanuts	R1	Sample/decision aggregation	Cumulative learning is relevant to lifecycle management, but pixel outputs require external decision validation.
Deng et al. [4]	ZEN/AFB_1_; non-imaging NIR transfer context	Ctx. R2	HSI/spatial evidence	Shows target-domain correction need; not a full HSI or cross-instrument deployment proof.
Zhang et al. [23]	AFB_1_ levels; portable VIS–NIR context	Ctx. R1	HSI/spatial and deployment evidence	Rapid portable screening context only; not unit-level AI-HSI evidence.

**Table 4:** Sensing platforms and their most credible food-safety roles.

Platform	Typical Contribution	Key Constraint	Most Credible Role
VIS–NIR reflectance	Surface color, defects, pigments, and moisture-related variation	Sensitive to illumination and surface presentation	Rapid screening of intact units
SWIR reflectance	Bond-related information from water, proteins, lipids, starch, and structure	High-dimensional data; instrument and moisture effects	Matrix-aware classification or regression
Fluorescence HSI	Enhanced response from fungal or endogenous fluorophores	Signal overlap and dependence on excitation conditions	Targeted screening and data fusion
Raman imaging	Complementary molecular fingerprint information	Weak signals and longer acquisition times	Focused verification or research-stage applications
Multispectral imaging	Selected stable wavelength bands	Depends on prior feature selection	Low-latency sorting and portable deployment

Abbreviations: VIS–NIR, visible and near-infrared; SWIR, short-wave infrared; HSI, hyperspectral imaging.

**Table 5:** Selected illustrative studies from the combined evidence map, stratified by hazard, reference anchor, contamination design, and translation maturity.

Study	Matrix and Target	Reference/Sensor-Model	Critical Deployment Interpretation
Alisaac et al. [16]	Wheat kernels/flour; Fusarium and DON	qPCR and LC–MS/MS; VIS–NIR and SWIR HSI	Links optical responses to biological and chemical endpoints, but correlation is not direct toxin detection.
Kim et al. [10]	Ground maize; aflatoxins	HPLC; naturally contaminated ground maize; VIS–NIR, SWIR, fluorescence, and Raman HSI; LDA/SVM	In the authors’ ground-maize data set, some models reported 95.7% classification accuracy and no false negatives at the study-specific 10 µg/kg cut-off. This is not a universal legal threshold or an external deployment result.
Kim et al. [7]	Ground maize; aflatoxin, fumonisin, co-contamination	HPLC and LC–MS/MS; naturally contaminated ground maize; VIS–NIR, SWIR, fluorescence HSI; PLS-DA/SVM	Study-specific multi-hazard classification reached a reported 95.7% best result. External, moving-line, and cross-site validation were not reported.
Wang et al. [8]	Peanuts; aflatoxin risk	Natural contamination; HSI with Vision Transformer fusion	Authentic peanuts support relevance, but the reported validation accuracy (92.6%) and recall (94.4%) remain study-specific and do not establish transfer or operation at line speed.
Chu et al. [9]	Individual maize kernels; AFB_1_ classes	Field inoculation; SWIR HSI with SVM	Validation accuracy of 82.5% illustrates the limits of early kernel-level screening.
Nadimi et al. [17]	Wheat; Fusarium damage and DON	NIR-HSI with 3NN	Fusarium-damage accuracy exceeded DON accuracy, reinforcing target-specific evaluation.
Yao et al. [3]	Individual maize kernels; AFB_1_ prediction	Inoculated kernels and reference analysis; dual-wavelength fluorescence HSI; fusion/PLS	Decision-level fusion improved prediction, while contamination heterogeneity remained consequential.
Deng et al. [4] ^†^	Wheat ZEN and peanut AFB_1_	FT-NIR and portable NIR datasets; transfer learning	Direct cross-task transfer was unreliable; target-domain correction improved task-specific prediction. The study did not establish fully validated cross-instrument deployment.
Zhang et al. [18]	Corn grits; ZEN and OTA	HSI; predominantly artificial contamination with a small natural-validation subset; CNN–GRU–Attention; SHAP	Provides early OTA-oriented, interpretable evidence. Predominantly controlled contamination and a small natural subset preclude any claim of routine deployment readiness.

Abbreviations: AFB_1_, aflatoxin B_1_; DON, deoxynivalenol; ZEN, zearalenone; qPCR, quantitative polymerase chain reaction; HPLC, high-performance liquid chromatography; LC–MS/MS, liquid chromatography–tandem mass spectrometry; VIS–NIR, visible and near-infrared; SWIR, short-wave infrared; HSI, hyperspectral imaging; LDA, linear discriminant analysis; SVM, support vector machine; PLS-DA, partial least squares discriminant analysis; 3NN, three-nearest-neighbor classifier; PLS, partial least squares; FT-NIR, Fourier-transform near-infrared. ^†^ Contextual non-imaging NIR study retained solely to inform transferability or portability; not interpreted as equivalent to HSI evidence.

**Table 6:** Comparative methodological concepts used to interpret validation strategies, datasets, algorithms, and decision relevance.

Concept	Categories Compared	Interpretive Purpose	Example Implication
Target endpoint	Fungal status; toxin concentration; threshold class; co-contamination; contextual transferability	Prevents fungal-status models from being interpreted as direct mycotoxin quantification.	A mold-status classifier can support early warning but not final toxin decisions.
Measurement unit	Pixel; kernel/nut; powder; bulk sample; lot; dataset	Links model output to the food-industry decision unit.	Kernel-level outputs are relevant to sorting; bulk values are relevant to lot triage.
Dataset design	Natural; inoculated; spiked; mixed; secondary/contextual	Shows whether spectral variation reflects real contamination complexity.	Artificial contamination supports feasibility but rarely supports deployment claims.
Reference method	HPLC/LC–MS/MS; ELISA; fungal DNA; spore count; visual grade; contamination proportion	Determines whether the model is aligned with toxin risk or another biological endpoint.	A bulk HPLC label cannot automatically validate single-kernel toxin classification.
Algorithm family	Chemometrics; SVM/LDA/RF/kNN; CNN/transformer; transfer learning; fusion models	Avoids equating algorithm complexity with food-safety readiness.	A simple model with external validation may be more useful than a complex internally validated model.
Validation strategy	Random split; cross-validation; independent holdout; external-domain test; transfer test; pilot line	Distinguishes model development from translational evidence.	Random splits do not establish performance across seasons, devices, or lines.
Decision output	Accept; hold; sort/reject; resample; refer to confirmation; monitor drift	Connects prediction to a protective food-safety action.	Indeterminate predictions should trigger hold or confirmation, not forced acceptance.

**Table 7:** Safety-critical performance measures for AI-enabled hyperspectral food-safety systems.

Measure	What It Reveals	Food-Safety Interpretation
Sensitivity/recall	Proportion of true positives identified	Primary protection against false-negative release decisions
Specificity	Proportion of true negatives identified	Controls unnecessary holds and product loss
Precision	Proportion of predicted positives that are correct	Reflects burden of unnecessary confirmation or rejection
F1-score	Balance of precision and recall	Useful for imbalanced classes, but not a substitute for false-negative counts
ROC-AUC	Ranking ability across thresholds	Must be complemented by performance at the selected action limit
RMSEP and bias	Magnitude and direction of concentration error	Essential for regression, especially near regulatory limits
RPD and R^2^	Relative fit and predictive spread	Can appear strong when the concentration range is broad
Confusion matrix/error profile	Class-specific mistakes and threshold failures	Required to judge the safety of accept, hold, and reject decisions
PPV and NPV	Correctness of positive and negative decisions at expected field prevalence	Essential because screening value changes when positives are rare or clustered.
Precision-recall curve	Trade-off between recall and precision under imbalance	More informative than ROC-AUC when contaminated samples are uncommon.
Confidence intervals	Uncertainty around performance estimates	Prevents overinterpreting small or narrow validation sets.
Threshold/probability calibration	Agreement between predicted risk and observed frequency	Supports calibrated hold, release, or confirmation thresholds.
Decision-curve or cost-sensitive analysis	Net benefit or cost under alternative thresholds	Links model output to food-safety and economic consequences.
Indeterminate/abstention rate	Frequency of samples routed to repeat imaging or confirmation	Required when uncertainty-aware systems avoid unsafe automated decisions.

Abbreviations: ROC-AUC, receiver operating characteristic area under the curve; RMSEP, root mean square error of prediction; RPD, residual predictive deviation; R^2^, coefficient of determination.

**Table 8:** Requirements for explainability and auditable AI-enabled hyperspectral decisions.

Requirement	Practical Evidence	Interpretive Caution
Feature importance	Stable selected wavelengths across resampling, batches, and external sets	Selected bands may represent moisture, background, or other correlated factors
Local explanation	SHAP or LIME explanation for borderline or rejected samples	Local explanations can vary with correlated variables and perturbation choices
Operational audit trail	Input file, sample/lot ID, model version, calibration record, threshold, confidence/uncertainty flag, and operator action	Mandatory for high-stakes use even when mechanistic explanation is incomplete
XAI faithfulness check	Stability, randomization, perturbation, or ablation tests for SHAP, LIME, saliency, or attention maps	Post hoc explanations can be unstable or unfaithful; they should not be accepted uncritically
Use-case dependence	Define whether explanations are needed for scientific interpretation, operator trust, model debugging, or regulatory audit	Not every task requires the same level of mechanistic explanation, but all need traceability
Spatial attribution	Saliency, Grad-CAM, or pixel maps linked to the trained unit	A map is not evidence of toxin distribution unless pixel labels exist
Mechanistic interpretation	Association with plausible O–H, C–H, N–H, water, protein, starch, lipid, or fungal-biomass responses	Plausibility does not establish a toxin-specific optical signal
Audit trail	Recorded model version, sensor setting, threshold, confidence, and operator action	Auditability fails without controlled change management
Human review	Hold-and-confirm rule for high-uncertainty or out-of-domain samples	Experts should not override output without documented rationale

Abbreviations: SHAP, Shapley additive explanations; LIME, local interpretable model-agnostic explanations; Grad-CAM, gradient-weighted class activation mapping.

**Table 9:** Sources of domain shift and corresponding model-lifecycle responses.

Source of Change	Likely Spectral Consequence	Lifecycle Response
Cultivar, origin, season, storage	Matrix composition, moisture, texture, and fungal ecology differ	Cross-batch and temporal validation; broaden calibration set
Artificial versus natural contamination	Different localization, growth stage, and matrix response	Record contamination mechanism; validate with authentic material
Instrument or illumination	Wavelength response, noise, exposure, and geometry shift	Reference standards, harmonization, transfer set, recalibration
Process conditions	Conveyor motion, orientation, dust, temperature, and overlap	Pilot-line stress testing and out-of-distribution alerts
Prevalence and threshold	Predictive values and false-negative burden change	Review action limits and confirmatory-test outcomes
Model update	New data may improve one domain but degrade another	Version control, retained benchmark data, controlled retraining

**Table 10:** Proposed Food–AI Deployment-Readiness Framework: core evidence domains.

Domain	Decision Question	Minimum Evidence for Progression
Analytical validity	Does the system address a clearly defined hazard using suitable ground truth?	Traceable reference method; optical/reference-unit alignment; relevant concentration range; explicit threshold; sampling plan; recovery/matrix-effect or method-performance information; LOD/LOQ; replicate or precision evidence; and uncertainty near the threshold where relevant.
Algorithmic robustness	Is the reported performance credible and safety-aware?	Leakage-controlled split; class-specific metrics; false-negative analysis; PPV/NPV at plausible prevalence where applicable; confidence intervals; calibration/threshold evidence; and independent holdout.
Explainability and auditability	Can a prediction be inspected and reconstructed?	Audit trail is mandatory: model version, calibration record, input traceability, uncertainty flag, threshold, and operator action. Post hoc explanation is desirable/use-case dependent and should be checked for stability/faithfulness.
Transferability and maintenance	Can performance withstand routine sources of domain shift?	Cross-batch or external evidence, calibration checks, drift monitoring, and update protocol.
Operational feasibility	Can the system work at the required speed and in the target environment?	Mandatory for R4+: quantitative throughput, latency, product-handling conditions, calibration frequency, and confirmation workflow. Desirable supporting evidence includes reject-actuation accuracy, downtime, environmental tolerance, computing load, and cost of ownership.
Decision integration	Does output trigger a defined protective action?	HACCP-compatible use case, accept/hold/segregate rules, confirmation pathway, and traceability.

**Table 11:** Provisional F-AIDRF translation stages and minimum evidence expectations.

Stage	Descriptive Classification	Minimum Evidence Expectation
R0	Not deployment-assessable	Unclear target, inadequate reference method, or insufficient reporting.
R1	Laboratory proof-of-concept	Controlled data and internal validation; limited evidence of operational robustness.
R2	Analytical screening candidate	Defined action threshold, independent holdout, class-specific metrics, and false-negative analysis. Mandatory: traceable reference, threshold-specific errors, and no unresolved unit mismatch for claimed decision.
R3	Externally validated decision-support candidate	Cross-batch, temporal, cultivar, regional, or target-domain validation; transfer strategy. Mandatory: all six domains must meet the R3 decision-support criteria or the system remains R2.
R4	Pilot-line implementation candidate	Moving-product evaluation, throughput and latency data, operator workflow, and hold or reject mechanism. Mandatory: quantitative throughput/latency/product-handling evidence.
R5	Evidence of routine industrial implementation (not regulatory approval)	Ongoing drift surveillance, controlled updates, traceability, cybersecurity, human oversight, and a confirmatory-testing pathway.

**Table 12:** Food-industry applications of F-AIDRF and evidence required before use.

Industry Use Case	Decision Supported	F-AIDRF Contribution	Evidence Required Before Routine Use
Incoming lot reception	Accept, hold, resample, or prioritize confirmatory testing.	Clarifies threshold logic, false-negative tolerance, reference alignment, and traceability.	Natural lot-level data, field-prevalence metrics, PPV/NPV, indeterminate rule, and confirmatory-referral protocol.
Kernel or nut sorting	Remove high-risk individual units before blending or processing.	Links spectral unit, reference unit, reject actuation, and product-overlap evidence.	Single-unit validation, actuation accuracy, conveyor velocity, latency, overlap rate, and post-sort verification.
Storage and processing early warning	Trigger inspection, drying, segregation, or intensified sampling.	Separates early fungal-status alerts from direct toxin claims.	Time-course validation, environmental tolerance, calibration checks, and confirmation of alert thresholds.
Laboratory triage	Prioritize samples for HPLC, LC–MS/MS, ELISA, or retesting.	Defines AI-HSI as a screening and prioritization layer rather than a confirmatory substitute.	Reference-method alignment, uncertainty flags, confirmation workload analysis, and audit trail.
Technology procurement and vendor evaluation	Compare commercial or prototype systems before purchase or pilot testing.	Provides a structured checklist for evidence missing from vendor accuracy claims.	Independent validation, maintenance plan, version control, operator training, total cost of ownership, and service/calibration documentation.
Post-deployment monitoring	Detect drift, recalibrate, suspend use, or update the model.	Connects model lifecycle to HACCP-compatible records and confirmatory checks.	Routine QC spectra, drift limits, update protocol, revalidation trigger, and documented operator action.

## Data Availability

No original experimental data were generated in this study. Review-derived data were created through evidence extraction, coding, and F-AIDRF stage assignment. The extraction sheet, coding definitions, selection record, study-level ratings, and framework-assignment rationale are provided in the Supplementary Materials and the reusable spreadsheet file accompanying the article.

## Supplementary Materials

The following supporting information can be downloaded at: https://www.mdpi.com/article/10.3390/foods15162860/s1, Table S1: Study-level F-AIDRF critical-appraisal profiles, optical/reference-unit alignment, contamination origin, validation/leakage-control status, failed readiness gates, and descriptive readiness-stage coding for the combined 20-study evidence map, consisting of 17 spectral-imaging entries and three contextual non-imaging NIR entries. (A) Study identification, evidence category, target, and optical/reference-unit alignment. (B) Validation/leakage-control coding, transfer/operation evidence, and descriptive F-AIDRF stage. (C) Failed readiness gate and rationale for each study-level F-AIDRF assignment; Table S2: Literature-identification logic, eligibility criteria, query blocks, screening record, exclusion categories, and evidence-boundary decisions; Table S3: F-AIDRF structured checklist: mandatory versus desirable criteria and adjudication rules; Table S4: Reusable extraction fields and coding definitions.
