# Supplementary material for: A Preliminary Food–AI Readiness Framework for AI-Enabled Hyperspectral Systems in Fungal and Mycotoxin Risk Management: A Critical Narrative Review
Source: Foods. 2026 Aug 17;15(16):2860. doi: 10.3390/foods15162860 (PMC13512283; doi:10.3390/foods15162860)
Supplement: Supplementary file 1 [file foods-15-02860-s001.zip › foods-4447248-supplementary.pdf]

Supplementary Materials

Note: Condensed versions of the evidence-identification record and the study-level readiness profile are provided in the main manuscript as Tables 1 and 3. The supplementary file retains the full reusable appraisal profiles, search logic, coding manual, and data dictionary for transparency and reuse.

A Preliminary Food–AI Readiness Framework for AI-Enabled Hyperspectral Systems in Fungal and Mycotoxin Risk Management: A Critical Narrative Review

Table S1: Study-level F-AIDRF critical-appraisal profiles, optical/reference-unit alignment, contamination origin, validation/leakage-control status, failed readiness gates, and descriptive readiness-stage coding for the combined 20-study evidence map, consisting of 17 spectral-imaging entries and three contextual non-imaging NIR entries.

Table S1A. Study identification, evidence category, target, and optical/reference-unit alignment.

| Study | Category | Matrix / target | Optical unit | Reference / label unit | Unit alignment and mismatch note | Contamination origin |
|---|---|---|---|---|---|---|
| Yao et al. [3] | Spectral imaging | Maize / $AFB_1$ | Single-kernel fluorescence HSI | Kernel-level $AFB_1$ reference | Relatively strong unit alignment; inoculation limits natural inference | Controlled / inoculated |
| Williams et al. [6] | Spectral imaging | Pistachio / aflatoxin infection | Pistachio HSI images | Aflatoxin infection labels | Aligned for sample classification; legal-threshold use not established | Study-specific material |
| Kim et al. [7] | Spectral imaging | Ground maize / aflatoxin, fumonisin, co-contamination | Ground-maize HSI | HPLC/LC–MS/MS class labels | Aligned for ground-sample classification; not individual-kernel detection | Naturally contaminated |
| Wang et al. [8] | Spectral imaging | Peanuts / aflatoxin risk | Peanut HSI images | Naturally contaminated peanut labels | Relevant natural matrix; output remains study-specific classification | Naturally contaminated |
| Chu et al. [9] | Spectral imaging | Maize kernels / $AFB_1$ classes | Single-kernel SWIR-HSI | Kernel $AFB_1$ classes | Supports kernel screening; field inoculation limits natural inference | Field inoculated |
| Kim et al. [10] | Spectral imaging | Ground maize / aflatoxins | VIS–NIR, SWIR, fluorescence, Raman HSI | HPLC aflatoxin values | Strong for ground-sample screening at study-specific cutoff; not universal legal threshold | Naturally contaminated regulatory samples |
| Yang et al. [11] | Spectral imaging | Stored maize / mold status | VIS–NIR HSI | Fungal spore-count mold classes | Aligned for fungal status, not toxin concentration | Controlled storage/mold status |
| Siripatrawan and Makino [12] | Spectral imaging | Brown rice / fungal contamination | Bulk rice HSI | Contamination proportion and volatile context | Aligned for contamination proportion; not mycotoxin quantification | Controlled mixing |
| Zhou et al. [13] | Spectral imaging | Maize kernels / $AFB_1$ groups | Single-kernel NIR-HSI | $AFB_1$ concentration classes | Kernel-level alignment; independent-test drop shows external challenge | Study-specific contaminated kernels |
| Bailly et al. [14] | Contextual non-imaging NIR | Ground maize / aflatoxin thresholds | NIR spectra without imaging | HPLC–MS values and threshold classes | Relevant to threshold screening but not spatial sorting | Naturally contaminated surveillance samples |
| Kabir et al. [15] | Spectral imaging | Almonds / $AFB_1$ | Almond HSI images | $AFB_1$ class labels | Aligned for almond classification; natural evidence limited | Controlled / laboratory material |
| Alisaac et al. [16] | Spectral imaging | Wheat / Fusarium and DON | VIS–NIR/SWIR HSI | qPCR fungal DNA and LC–MS/MS toxins | Multi-endpoint reference; fungal and toxin endpoints must be separated | Inoculated Fusarium infection |
| Nadimi et al. [17] | Spectral imaging | Wheat / Fusarium damage and DON | NIR-HSI | Fusarium-damage and DON labels | Shows mismatch between fungal damage and DON detection | Field-derived/natural wheat material |

| Study | Category | Matrix / target | Optical unit | Reference / label unit | Unit alignment and mismatch note | Contamination origin |
|---|---|---|---|---|---|---|
| Zhang et al. [18] | Spectral imaging | Corn grits / ZEN and OTA | HSI | ZEN and OTA concentrations/classes | Relevant OTA/ZEN reference; artificial dominance limits natural inference | Predominantly artificial; small natural subset |
| Kandpal et al. [19] | Spectral imaging | Corn kernels / $AFB_1$ | SWIR-HSI | $AFB_1$ reference classes/concentrations | Kernel-level $AFB_1$ relevance; early design limits deployment inference | Controlled/study-specific contamination |
| Mishra et al. [20] | Spectral/MSI | Almonds / aflatoxin | HSI-derived bands and MSI prototype | $AFB_1$ contamination classes | Useful sorting concept; artificial contamination limits natural alignment | Artificial/controlled |
| Zhang et al. [21] | Spectral imaging | Maize / $AFB_1$ quantification | HSI with attention network | $AFB_1$ quantitative reference | Aligned within dataset; threshold evidence limited | Controlled/study-specific samples |
| Zhao et al. [22] | Spectral imaging | Peanuts / aflatoxin pixel-level detection | Pixel-level peanut HSI | Aflatoxin labels from study datasets | Pixel-level output requires aggregation to sample decision | Controlled/multi-source datasets |
| Deng et al. [4] | Contextual non-imaging NIR | Wheat ZEN and peanut $AFB_1$ | FT-NIR and portable NIR spectra | Chemical concentrations | Useful transfer logic; not HSI/spatial sorting evidence | Existing spectral datasets |
| Zhang et al. [23] | Contextual non-imaging NIR | Corn / $AFB_1$ levels | Portable VIS–NIR spectra | $AFB_1$ labels | Portable screening context; not unit-level HSI evidence | Study-specific corn samples |

Table S1B. Validation/leakage-control coding, transfer/operation evidence, and descriptive F-AIDRF stage.

| Study | Validation and leakage-control status | Transfer / operational evidence | Descriptive F-AIDRF stage |
|---|---|---|---|
| Yao et al. [3] | Study-specific validation; leakage-control details depend on reported splitting | Data fusion evidence; no routine line deployment | R1 |
| Williams et al. [6] | Internal validation; recall relevant to false-negative control | No line-level deployment | R1 |
| Kim et al. [7] | Internal study-specific validation; no cross-site or moving-line test | Multi-hazard evidence; no pilot line | R2 |
| Wang et al. [8] | Validation reported; transfer/leakage status limited by reported design | No line speed or cross-site deployment | R2 |
| Chu et al. [9] | Internal validation; no external deployment test | Early kernel-level sorting relevance | R1 |
| Kim et al. [10] | Internal threshold evaluation; no false negatives at study-specific cutoff in this dataset | Laboratory screening workflow; no moving-line deployment | R2 |
| Yang et al. [11] | Study-specific train/test validation; no cross-site validation | Early warning potential | R1 |
| Siripatrawan and Makino [12] | Internal validation; leakage-control not fully inferable | Early contamination screening | R1 |
| Zhou et al. [13] | Independent-test performance reported; broader transfer absent | Screening candidate; no line evidence | R1 |
| Bailly et al. [14] | Threshold classification reported; no HSI leakage issue | Regulatory-screening context only | Contextual R2 |
| Kabir et al. [15] | Validation reported; external natural validation not established | No industrial validation | R1 |
| Alisaac et al. [16] | Study-specific modeling; cross-domain evidence limited | Mechanistic relevance; no deployment | R1 |
| Nadimi et al. [17] | Internal validation; DON sensitivity lower than damage classification | No deployment evidence | R1 |
| Zhang et al. [18] | Internal and limited natural validation; leakage-control requires caution | XAI wavelength evidence; no deployment | R1 |
| Kandpal et al. [19] | Internal validation; no external transfer | Early feasibility only | R1 |
| Mishra et al. [20] | Validation reported; natural validation absent/limited | Throughput-oriented MSI prototype | R2 |
| Zhang et al. [21] | Internal regression validation; external/pilot evidence limited | No line-level evidence | R1 |
| Zhao et al. [22] | Cumulative learning explored; forgetting addressed conceptually | Model-lifecycle relevance; no routine deployment | R1 |
| Deng et al. [4] | Direct transfer poor; target-domain recalibration improved prediction | Contextual calibration-transfer evidence only | Contextual R2 |
| Zhang et al. [23] | Rapid classification protocol; independent deployment evidence limited | Portable context only | Contextual R1 |

Note: R1 = laboratory proof-of-concept; R2 = analytical screening candidate. The combined denominator is 20 study entries, but the HSI-equivalent stage count uses the 17 spectral-imaging studies; the three contextual non-imaging NIR entries are flagged separately and are not treated as HSI-equivalent evidence. Coding is single-author, conservative, and based on reported information only.

Table S2. Literature-identification logic, eligibility criteria, query blocks, screening record, exclusion categories, and evidence-boundary decisions.

| Item | Description |
|---|---|
| Review type | Critical narrative review with structured evidence appraisal; not a systematic review, meta-analysis, PRISMA review, or exhaustive census. |
| Coverage date | Candidate records and bibliographic/regulatory records were checked through 1 July 2026. |
| Concept block 1: sensing | hyperspectral OR multispectral OR "spectral imaging" OR fluorescence OR Raman OR VIS–NIR OR SWIR OR "near-infrared" |
| Concept block 2: modeling | AI OR "artificial intelligence" OR "machine learning" OR "deep learning" OR chemometrics OR "transfer learning" OR "calibration transfer" OR "explainable AI" OR SHAP OR LIME |
| Concept block 3: hazard | fungal OR mold OR mould OR Aspergillus OR Fusarium OR Penicillium OR aflatoxin* OR fumonisin* OR deoxynivalenol OR DON OR zearalenone OR ZEN OR ochratoxin OR OTA OR patulin OR mycotoxin* OR co-contamination |
| Concept block 4: matrix | food OR feed OR grain OR cereal OR maize OR corn OR wheat OR peanut OR almond OR pistachio OR rice OR nut OR oilseed OR flour OR processed food |
| Core Boolean logic | (block 1) AND (block 2) AND (block 3) AND (block 4), with targeted substitutions for individual toxins and matrices. |
| Primary inclusion | Food/feed spectral-imaging studies using an AI, machine-learning, deep-learning, or chemometric model and an identifiable reference target. |
| Contextual inclusion | Non-imaging NIR studies retained only for portability, calibration transfer, threshold-screening, or regulatory-context discussion. |
| Exclusion rules | Non-food matrices; general quality studies without fungal or mycotoxin target; descriptive spectroscopy without predictive model; no identifiable reference target; no peer-reviewed record; non-English records. |
| Patulin boundary | Patulin was included in query logic, but no patulin-specific AI-HSI primary study was retained in this focused corpus. This is a corpus boundary statement, not a universal absence claim. |
| Regulatory/context sources | Two EU legal sources, one Codex standard, and one FDA guidance document were used as context for thresholds and confirmatory analysis. They were not primary AI-HSI evidence. |
| F-AIDRF coding rule | A study could not progress beyond R1 without traceable reference alignment; beyond R2 without independent or cross-domain validation evidence; beyond R3 without transfer/external decision-support evidence; or beyond R4 without pilot-line/moving-product and governance evidence. |

Table S1C. Failed readiness gate and rationale for each study-level F-AIDRF assignment.

| Study | Assigned stage | Primary failed gate | Assignment rationale |
|---|---|---|---|
| Yao et al. [3] | R1 | External/pilot-line evidence | Controlled inoculation and study-specific validation. |
| Williams et al. [6] | R1 | External/pilot-line evidence | Recall evidence without deployment/transfer testing. |
| Kim et al. [7] | R2 | External decision-support evidence | Natural multi-hazard data but no cross-site/line validation. |
| Wang et al. [8] | R2 | External transfer and line evidence | Natural contamination but study-specific validation. |
| Chu et al. [9] | R1 | Natural/external validation | Field inoculation and no line validation. |
| Kim et al. [10] | R2 | External/pilot-line evidence | Natural samples and threshold errors, but not operational deployment. |
| Yang et al. [11] | R1 | Toxin/reference alignment | Fungal-status label, not toxin concentration. |
| Siripatrawan and Makino [12] | R1 | Mycotoxin/reference alignment | Contamination proportion, not toxin concentration. |
| Zhou et al. [13] | R1 | External robustness | Independent-test performance lower; broader transfer absent. |
| Bailly et al. [14] | Contextual R2 | HSI/spatial evidence | Non-imaging threshold-screening context. |
| Kabir et al. [15] | R1 | Natural/external validation | Laboratory-stage almond classification. |
| Alisaac et al. [16] | R1 | Decision-level validation | No deployment/cross-domain evidence. |
| Nadimi et al. [17] | R1 | DON sensitivity/deployment | DON evidence weaker than Fusarium-damage evidence. |
| Zhang et al. [18] | R1 | Natural/external validation | Mainly artificial contamination; limited natural subset. |
| Kandpal et al. [19] | R1 | External/pilot-line evidence | Early feasibility only. |
| Mishra et al. [20] | R2 | Natural validation | Throughput prototype but artificial contamination. |
| Zhang et al. [21] | R1 | External threshold validation | Study-specific regression evidence. |
| Zhao et al. [22] | R1 | Sample/decision aggregation | Pixel outputs need decision-level aggregation. |

| Study | Assigned stage | Primary failed gate | Assignment rationale |
|---|---|---|---|
| Deng et al. [4] | Contextual R2 | HSI/spatial evidence | Non-imaging transfer context. |
| Zhang et al. [23] | Contextual R1 | HSI/spatial evidence | Portable VIS–NIR context only. |

Table S3. F-AIDRF structured checklist: mandatory versus desirable criteria and adjudication rules.

Note: For independent validation, this checklist should be applied by multiple trained assessors to an external study set not used in the present framework derivation. Agreement should be quantified using kappa or weighted/ordinal agreement statistics, and disagreements should be adjudicated with reasons recorded before the checklist is revised. Mandatory criteria determine whether a study can progress to a higher readiness stage, whereas desirable evidence strengthens the interpretation but is not, by itself, required in every use case. Auditability is mandatory for high-stakes use; post hoc XAI is desirable/use-case dependent; and quantitative operational evidence is mandatory only for R4+ pilot-line claims.

| Domain | Mandatory criteria for progression | Desirable evidence | Examples | Adjudication rule |
|---|---|---|---|---|
| Analytical validity | Defined endpoint; suitable reference; aligned optical/reference unit; relevant concentration or threshold; minimum method performance information. | Recovery, matrix effects, LOD/LOQ, replicates, CRM/QC material, uncertainty near threshold. | HPLC/LC–MS/MS value aligned to imaged sample; qPCR clearly treated as fungal-status endpoint. | Unclear or mismatched reference unit prevents advancement beyond R1/R2 depending on intended claim. |
| Algorithmic robustness | Leakage-aware split; class-specific metrics; false-negative reporting; independent holdout for R2+. | PPV/NPV at expected prevalence; confidence intervals; PR curves; calibration plots. | Batch-level split; external natural test set; threshold error table. | If split unit is unclear, leakage control is coded unclear and stage is capped conservatively. |
| Explainability/auditability | Audit trail for input, model version, calibration status, threshold, uncertainty flag, and operator action. | Stable wavelength/spatial explanation; SHAP/LIME/saliency faithfulness checks. | Wavelength attribution consistent with known chemistry; logged operator override. | Auditability is mandatory for high-stakes use; post hoc XAI is desirable, use-case dependent, and should not be treated as a universal mandatory criterion. |
| Transferability/maintenance | Evidence beyond a single random split for R3+; update or recalibration protocol. | Cross-season, cross-cultivar, cross-instrument, or target-domain correction tests. | External batch validation; drift-monitoring schedule. | No external/domain evidence means system cannot exceed R2. |
| Operational feasibility | Quantitative use-context for R4+: throughput, latency, product handling, calibration frequency, and confirmation workflow. | Reject-actuation accuracy, downtime, environmental tolerance, computing load, cost of ownership. | Moving-product trial; conveyor speed; confirmation workload estimate. | No quantitative operation data means no R4/pilot-line readiness claim; additional cost, downtime, environmental-tolerance, and computing-load evidence is desirable rather than universally mandatory. |
| Decision integration | Defined accept/hold/sort/reject/resample/confirmatory pathway; role within HACCP or QA workflow. | Decision-curve or cost-sensitive analysis; indeterminate pathway; stakeholder-defined thresholds. | Screen-sort-confirm workflow; hold for HPLC/LC–MS/MS. | If output is only a model score without defined action, decision integration is insufficient. |

Table S4. Reusable extraction fields and coding definitions.

| Field | Coding definition | Allowed/expected entries |
|---|---|---|
| Study ID | First author and citation number used in the main manuscript. | Author et al. [number] |
| Evidence category | Whether evidence is spectral imaging, spectral/MSI, or contextual non-imaging NIR. | Spectral imaging; Spectral/MSI; Contextual non-imaging NIR |
| Endpoint category | Primary target endpoint. | Fungal status; toxin threshold; toxin regression; co-contamination; contextual transfer/portable screening |
| Optical unit | Unit represented by the HSI/NIR input. | Pixel; kernel; nut; powder/ground sample; bulk lot; spectrum |
| Reference unit | Unit represented by chemical/molecular/microbiological/visual reference label. | Kernel; pooled sample; ground sample; lot; visual class; DNA/spore count |
| Contamination origin | Source and realism of contamination. | Natural; field inoculated; artificial/spiked; controlled; mixed; unclear |

| Field | Coding definition | Allowed/expected entries |
|---|---|---|
| Validation design | How model was tested. | Internal CV; random holdout; independent test; external batch; transfer test; pilot/moving-line |
| Leakage-control status | Whether related observations were kept independent across splits. | Adequate; partially reported; unclear; not applicable |
| Failed readiness gate | First gate that prevents higher F-AIDRF stage. | Reference alignment; false-negative/threshold evidence; external validation; transferability; operation; governance |
| Readiness stage | Provisional descriptive F-AIDRF stage. | R0; R1; R2; R3; R4; R5; Contextual R1/R2 |
